# Validation of *Fuscoporia* (*Hymenochaetales*, *Basidiomycota*) ITS sequences and five new species based on multi-marker phylogenetic and morphological analyses

**DOI:** 10.1186/s43008-023-00117-6

**Published:** 2023-06-28

**Authors:** Yoonhee Cho, Dohye Kim, Yoongil Lee, Juhwan Jeong, Shahid Hussain, Young Woon Lim

**Affiliations:** 1 School of Biological Sciences and Institute of Microbiology, Seoul National University, Seoul, Republic of Korea; 2 Centre for Plant Sciences and Biodiversity, University of Swat, Swat, Khyber Pakhtunkhwa Pakistan

**Keywords:** Annotation, Five new taxa, *Hymenochaetaceae*, ITS, Molecular identification, *Phellinus*

## Abstract

Although there is a continuous increase in available molecular data, not all sequence identities in public databases are always properly verified and managed. Here, the sequences available in GenBank for *Fuscoporia* (*Hymenochaetales*) were validated. Many morphological characters of *Fuscoporia* overlap among the species, emphasizing the role of molecular identification for accuracy. The identities of 658 *Fuscoporia* GenBank internal transcribed spacer (ITS) sequences were assessed using ITS phylogeny, revealing 109 (16.6%) misidentified and 196 (29.8%) unspecified sequences. They were validated and re-identified based on the research articles they were published in and, if unpublished, based on sequences from the type, type locality-derived sequences, or otherwise reliable sequences. To enhance the resolution of species delimitation, a phylogenetic assessment of a multi-marker dataset (ITS + nrLSU + *rpb2* + *tef1*) was conducted. The multi-marker phylogeny resolved five of the twelve species complexes found in the ITS phylogeny and uncovered five new *Fuscoporia* species: *F. dolichoseta*, *F. gilvoides*, *F. koreana*, *F. reticulata*, and *F. semicephala*. The validated ITS sequences in this study may prevent further accumulation of misidentified sequences in public databases and contribute to a more accurate taxonomic evaluation of *Fuscoporia* species.

**Supplementary Information:**

The online version contains supplementary material available at 10.1186/s43008-023-00117-6.

## INTRODUCTION

The nuclear ribosomal internal transcribed spacer (ITS) region is superior to other genetic regions as a DNA barcode for many fungal lineages because it is highly variable among species, easily amplified, and useful for phylogenetic inference (Schoch et al. 2012). Because of these characteristics, available fungal ITS data has continuously increased, but a substantial proportion remains insufficiently identified (Ryberg et al. 2009). Considering ITS as a universal DNA barcode for fungi (Schoch et al. 2012), unmediated ITS sequence uploads are problematic because of the limited number of type-derived sequences or annotated type-derived sequences available in the public database that would validate the matching sequence identities (Hofstetter et al. 2019). A previous study showed that a significant number of fungal DNA sequences had insufficient descriptions, with up to 20% of all entries in the International Nucleotide Sequence Database being incorrectly annotated with respect to taxonomy (Nilsson et al. 2006). For macrofungi, only 57% of all taxa in GenBank (Sayers et al. 2022) were found to be correctly named for species-level identification (Meiklejohn et al. 2019). On a smaller scale, *Bjerkandera* species reportedly had 10.5–13.8% misidentified GenBank ITS and nuclear large ribosomal subunit (nrLSU) sequences (Jung et al. 2014), whereas *Ganoderma* cf. *applanatum* and *G. lingzhi* each had 46% and 86% misidentified or ambiguously labelled ITS sequences, respectively (Jargalmaa et al. 2017).

A relatively high number of ITS GenBank sequences is present for another macro-fungal taxon, *Fuscoporia* (*Hymenochaetales*, *Basidiomycota*), as ITS is commonly used to identify *Fuscoporia* species, especially in non-taxonomic studies (Covino et al. 2019; Noji et al. 2021). Precise identification of *Fuscoporia* species is crucial because some species, such as *F. gilva* and *F. torulosa*, are being intensively studied for their medicinal effects (Deveci et al. 2019; Duong and Dang 2022). Incorrect species identification may cause confusion in establishing accurate species profiling for medicinal and biotechnological applications. However, *Fuscoporia* species are not easily classified and identified, as a wide range of morphological characters overlaps with those of many other *Hymenochaetaceae* species. Certain common characters include resupinate to pileate basidiomes that are mostly perennial, a dimitic hyphal system with encrusted hyphae at the dissepiment edge and tube cavities, presence of hymenial and mycelial setae, basidiospores which are smooth, thin-walled, and non-dextrinoid with shapes varying from allantoid, cylindrical, ellipsoid, ovoid, to subglobose (Fiasson and Niemelä, 1984; Wagner and Fischer 2001; Dai 2010). In addition, the ecological traits of *Fuscoporia* are not specific to the genus. Just like many *Hymenochaetaceae* species, those of *Fuscoporia* are found worldwide, causing white rot in the woods of both coniferous and deciduous trees (Panconesi et al. 1994; Luana et al. 2015), with some being parasitic (Spirin et al. 2014).

Owing to the lack of discriminatory morphological characters, some species of *Fuscoporia* have been classified in *Phellinus s. lat.* (Overholts 1953; Ryvarden and Johansen 1980; Larsen and Cobb-Poulle 1990), even though *Fuscoporia* was proposed as a legitimate genus in 1907 with *F. ferruginosa* as the type species (Murrill 1907). Fiasson and Niemelä (1984) reported *Fuscoporia* as a distinct taxonomic entity that could be distinguished from other *Hymenochaetaceae* by the presence of crystals in the generative hyphae and dark brown thick-walled hymenial setae. Later, *Fuscoporia* was recognized at the subgeneric level (Dai 1999). With the active use of molecular analysis and phylogenetic inference in fungal taxonomy, *Fuscoporia* has been revived as a distinct genus from *Phellinus s. str.* based on the nrLSU (Wagner and Fischer 2001, 2002). However, nrLSU phylogenetic analysis was found to be suitable only for differentiating genera and distantly related species in this part of *Hymenochaetales*. To address this issue, a combination of various DNA markers, such as ITS, nrLSU, RNA-polymerase II subunit (*rpb2*), and translation elongation factor 1 (*tef1*), has been used in phylogenetic studies of *Fuscoporia* (Chen and Dai 2019; Tchoumi et al. 2020; Wu et al. 2022). In addition to multi-marker analyses, the re-evaluation of *Fuscoporia* species through geographical distribution and micro-morphological characters has increased the resolution of species differentiation and identification, resulting in multiple re-classifications of species and the recognition of new species (Chen et al. 2019, 2020; Tchoumi et al. 2020).

The present study primarily aimed to validate *Fuscoporia* GenBank ITS sequences to highlight the substantial amount of data that requires revision and to explain the possible undesirable implications of misidentified sequences in future studies. Misidentified and unidentified *Fuscoporia* ITS sequences were re-identified to the species level based on type, type locality, or reliable published sequences that grouped together in a monophyletic clade in the ITS phylogeny. However, owing to the low resolution of ITS in species differentiation, there were some monophyletic clades that featured more than one distinct species. These species complexes were evaluated using a multi-marker (ITS + nrLSU + *rpb2* + *tef1*) phylogenetic inference since this approach has been used to resolve several species complexes before. Other methods to resolve these issues confidently are listed and suggested. Multi-marker analyses also revealed five new species that were supported as novel by the morphology and ecological data. Descriptions of the new *Fuscoporia* species are provided.

## MATERIALS AND METHODS

### Specimens studied

Fifty-two basidiomes of *Fuscoporia* were collected in the Republic of Korea and Pakistan from 2012 to 2019. They were stored as dry specimens at the Seoul National University Fungus Collection (SFC) and the University of Malakand herbarium. Images and notes on fresh basidiomes in the field, collection time, and location were recorded for each specimen.

### DNA extraction, PCR, and sequencing

Small pieces of tissue (approximately 1 × 1 cm) were isolated from each dried specimen using sterile forceps and scalpels. The isolated tissues were placed in 200 μl of 2 × Cetyltrimethyl ammonium bromide (CTAB) buffer and ground using a Bead Ruptor Elite (OMNI International, GA). Genomic DNA was extracted using the AccuPrep Genomic DNA Extraction Kit (Bioneer, Daejeon, Republic of Korea), following the manufacturer’s protocol.

Polymerase chain reaction (PCR) was performed with PCR Premix (Bioneer, Daejeon, Republic of Korea) using a C1000 thermal cycler (Bio-Rad, CA). The ITS region was amplified using primers ITS1F and ITS4B (Gardes and Bruns 1993) under the following conditions: initial denaturation at 95 °C for 5 min; 35 cycles of 95 °C for 40 s, 55 °C for 40 s, and 72 °C for 1 min; and a final extension at 72 °C for 5 min. The nrLSU region was amplified using primers LR0R and LR7 (Vilgalys and Hester 1990) under the same conditions as those used for ITS. The *rpb2* region was amplified with primers bRPB2-6F and bRPB2-7.1R (Matheny 2005) under the following conditions: initial denaturation at 94 °C for 2 min; 36 cycles of 94 °C for 45 s, 53 °C for 90 s, and 72 °C for 90 s; and a final extension at 72 °C for 10 min. The *tef1* region was amplified with primers EF595F and EF1160R (Kauserud and Schumacher 2001) under the following conditions: initial denaturation at 95 °C for 4 min; 35 cycles of 95 °C for 30 s, 55 °C for 30 s, and 72 °C for 1 min; and a final extension at 72 °C for 7 min.

All PCR products were verified by gel electrophoresis using a 1% agarose gel and Gel Doc XR (Bio-Rad, CA, USA). The PCR products were purified using the ExpinTM PCR Purification Kit (GeneAll Biotechnology, Seoul) following the manufacturer’s instructions. DNA sequencing was conducted at Bioneer (Daejeon, Republic of Korea) using an ABI 3730XL machine (Applied Biosystems, CA). All sequences were read using PCR primers. After a manual quality check for, e.g., chimeras and noise, the forward and reverse reads for each specimen were assembled using Geneious Prime 2022.0.2. The final sequences were submitted to GenBank (ITS: ON427761–ON427790, nrLSU: ON427791–ON427818, *rpb2*: ON464727–ON464731 and ON479778–ON479799, and *tef1*: ON479800–ON479821).

### ITS phylogenetic analysis

To construct the maximum likelihood (ML) phylogenetic tree for the ITS region, all GenBank sequences annotated as *Fuscoporia* and any sequence that closely matched the type-derived sequences through NCBI BLAST were retrieved. Sequences annotated as *Fuscoporia* but with low similarity to the rest of the *Fuscoporia* sequences were excluded (e.g., MH364762). The outgroup sequences included in the analysis were *Phellinidium fragrans* CBS 202.90 (NR_154284) and *Phellinidium ferrugineofuscum* Cui 10042 (KR350573). All reference and newly generated ITS sequences were aligned using MAFFT version 7 (Katoh and Standley 2013), and manual trimming was performed at the ends of the alignments (Additional file 1: Data S1). The ML tree was inferred using RAxML v.8.2.12 (Stamatakis 2014) with 1000 replications. The phylogenetic tree was used to re-identify misidentified or uncertain sequences based on type- or type locality-derived sequences. Sequences annotated with old synonyms have also been renamed. The reannotations were submitted to UNITE (Additional file 2: Table S1). Clades without a type- or type locality-derived sequence were annotated according to reliable published sequences with definite species identities. The topmost hit (Additional file 3: Table S2) and the top five hits (Additional file 4: Table S3) from the BLASTn results for all sequences are listed to address the accuracy of species annotation in the NCBI nr database. BLAST was performed on 2 December 2022, and the results were listed by the Per_ID values.

### Multi-marker phylogenetic analyses

To increase the resolution and reliability of the phylogenetic tree, the multi-marker phylogeny was assessed. Reliable reference sequences, including published and type-derived sequences, were downloaded from GenBank. Strains with sequences of at least three genetic regions available from ITS, nrLSU, *rpb2*, and *tef1* were selected, where possible, to increase the resolution of the multi-marker phylogenetic analyses (Table 1). All reference and newly generated sequences were aligned for each genetic region using MAFFT version 7 (Katoh and Standley 2013), and manual trimming was performed at the ends of the alignments. The four genetic regions were concatenated using Geneious Prime 2022.0.2.

**Table 1:** List of *Fuscoporia* specimens and GenBank accessions of ITS, nrLSU, *rpb2*, and *tef1* sequences analyzed

Species	Specimen voucher	Country (research article)	Accession				References	Remarks
			ITS	nrLSU	*rpb2*	*tef1*		
*F. acutimarginata*	Dai 15137	China	MH050751	MH050765	MN159384	MN848821	Chen and Dai (2019)	
	**Dai 16892**	**China**	**MH050752**	**MH050766**	**MH079393**	**MN848822**	Chen and Dai (2019)	
*F. ambigua*	Dai 16030	USA	MN816704	MN809994	MN848790	MN848803	Chen et al. (2020)	1. Annotated as *Fuscoporia* sp. in GenBank 2. Country annotated as China in GenBank for ITS
	**JV 0509/151**	**USA**	**MN816707**	**MN809996**	**MN848792**		Chen et al. (2020)	1. Misannotated as *Fuscoporia ferruginosa* in GenBank 2. Country annotated as China in GenBank for ITS
*F. americana*	JV 1209/100	USA	KJ940022	MG008467		MH636384	Du et al. (2020)	Annotated as *Fuscoporia* sp. in GenBank
*F. australasica*	Dai 15625	China	MN816726	MN810018	MN848775	MN848829	Chen et al. (2020)	Annotated as *Fuscoporia* sp. in GenBank
	**Dai 15636**	**China**	**MG008397**	**MG008450**	**MH079402**	**MH636408**	Chen and Dai (2019)	
	Dai 15659	China	MG008398	MG008451	MH079403	MH636409	Chen and Dai (2019)	
*F. australiana*	Dai 18587A	Australia	MN816723	MN810013	MN848765	MN848849	Chen et al. (2020)	
	Dai 18672	Australia	MN816703	MN810014	MN848766	MN848848	Chen et al. (2020)	Country annotated as USA in GenBank for ITS
	**Dai 18879**	**Australia**	**MN816705**	**MN810015**	**MN848767**	**MN848850**	Chen et al. (2020)	Country annotated as USA in GenBank for ITS
*F. bambusae*	**Dai 16599**	**Thailand**	**MN816711**	**MN809999**		**MN848808**	Chen et al. (2020)	Country annotated as China in GenBank for ITS
	Dai 16607	Thailand	MN816713	MN810000	MN848797	MN848809	Chen et al. (2020)	Country annotated as China in GenBank for ITS
	Dai 16615	Thailand	MN816715	MN810001		MN848810	Chen et al. (2020)	Country annotated as USA in GenBank for ITS
*F. bambusicola*	**Cui 8692**	**China**	**MN816739**	**MT032486**		**MN848813**	Chen et al. (2020)	
*F. callimorpha*	Doll 868		MN816701	MN809992		MN848840	Chen et al. (2020)	
	SFC20160128-06	Federated States of Micronesia	ON427767	ON427796	ON479778	ON479804	This study	
*F. caymanensis*	JV 1408/5	French Guiana	MW009110	MW009109			Vlasak et al. (2020)	
	**JV 1908/74**	**French Guiana**	**MT676832**	**MT676833**			Vlasak et al. (2020)	
*F. chinensis*	Cui 11209	China	MN121826	MN121767	MN159388		Chen and Dai (2019)	Annotated as *Fuscoporia gilva* in GenBank
	**Dai 15713**	**China**	**MN816721**	**MN810008**	**MN848771**	**MN848846**	Chen et al. (2020)	
	Dai 17282	China	MN816710	MN810009	MN848772	MN848847	Chen et al. (2020)	Country annotated as Thailand in GenBank for ITS
*F. contigua*	Dai 16025	USA	MG008401		MH079406	MH636386	Chen and Dai (2019)	
	JV 1204/22.3a,b-J	USA	KX961104	KY189104	MH079407		Chen and Yuan (2017)	Strain annotated as JV 1204/22 3 J for *rpb2*
*F. dolichoseta*	SFC20140723-58	Republic of Korea	ON427788	ON427816	ON479797	ON479820	This study	
	SFC20161006-16	Republic of Korea	ON427789	ON427817	ON479798	ON479821	This study	
	SFC20190731-26	Republic of Korea	ON427790	ON427818	ON479799		This study	
	**SFC20191015-23**	**Republic of Korea**	**ON427765**	**ON427795**	**ON464731**		**This study**	
*F. eucalypti*	Dai 18634A	Australia	MN816729	MN810020	MN848777	MN848830	Chen et al. (2020)	
	Dai 18783	Australia	MN816730	MN810021	MN848776	MN848832	Chen et al. (2020)	
	**Dai 18792**	**Australia**	**MN816731**	**MN810022**		**MN848831**	Chen et al. (2020)	
*F. ferrea*	Cui 11801	China	KX961101	KY189101		MN848823	Chen and Yuan (2017)	
	FP-133592-Sp	USA	KU139189	KU139259	KU139319	KU139379	Brazee (2015)	
	JV 1105/3 J	USA	MH050760	MH050770	MH079392		Chen and Dai (2019)	
	JV 1606/2.2-J	USA	KX961100	KY189100	MH079394	MH636402	Chen and Yuan (2017)	
*F. ferruginosa*	Cui 9244	China	MN816706	MN809995		MN848804	Chen et al. (2020)	
	Dai 13200	France	MN816702	MN809993	MN848793	MN848802	Chen et al. (2020)	
	JV 0408/28	Czech Republic	KX961103	KY189103		MH636397	Chen and Yuan (2017)	
	JV 1309/4	Slovakia	KX961102	KY189102	MH079405	MH636398	Chen and Yuan (2017)	
*F. gilva*	CMW47749	South Africa	MH599106	MH599129		MT108963	Tchoumi et al. (2020)	
	CMW48145	South Africa	MH599105	MH599130		MT108962	Tchoumi et al. (2020)	
	*JV 0709/75*	*USA*	*MN816720*	*MN810007*		*MN848852*	Chen et al. (2020)	Country annotated as Australia in GenBank for ITS
	*JV 1209/65*	*USA*	*MN816719*	*MN810006*		*MN848851*	Chen et al. (2020)	Country annotated as Singapore in GenBank for ITS
*F. gilvoides*	110N	Pakistan	ON427780	ON427809			This study	
	MUGBt	Pakistan	ON427781	ON427810	ON479791	ON479814	This study	
	MUKM-2	Pakistan	ON427782	ON427811	ON479792		This study	
	SFC20150702-23	Republic of Korea	ON427783		ON479793	ON479815	This study	
	SFC20160621-12	Republic of Korea	ON427784	ON427812	ON479794	ON479816	This study	
	SFC20160629-33	Republic of Korea	ON427785	ON427813	ON479795	ON479817	This study	
	**SFC20180426-12**	**Republic of Korea**	**ON427763**	**ON427793**	**ON464729**	**ON479802**	**This study**	
	SFC20180905-15	Republic of Korea	ON427786	ON427814	ON479796	ON479818	This study	
*F. insolita*	**JV 1208/5208-Spirin**	**Russia**	**MN816724**	**MN810016**		**MN848800**	Chen et al. (2020)	1. Strain annotated as Spirin 5208 in the reference article 2. Accessions for ITS and nrLSU switched in the reference article
*F. karsteniana*	**Dai 11403**	**China**	**MN816717**	**MN810003**	**MN848795**	**MN848807**	Chen et al. (2020)	
	Dai 15717	China	MN816718	MN810004		MN848805	Chen et al. (2020)	Country annotated as Australia in GenBank for ITS
	Dai 16552	China	MN816716	MN810002	MN848794	MN848806	Chen et al. (2020)	
*F. koreana*	SFC20150625-05	Republic of Korea	ON427776	ON427805	ON479787	ON479810	This study	
	SFC20150625-07	Republic of Korea	ON427777	ON427806	ON479788	ON479811	This study	
	**SFC20160726-93**	**Republic of Korea**	**ON427762**	**ON427792**	**ON464728**	**ON479801**	**This study**	
	SFC20171019-11	Republic of Korea	ON427778	ON427807	ON479789	ON479812	This study	
	SFC20180725-17	Republic of Korea	ON427779	ON427808	ON479790	ON479813	This study	
*F. latispora*	**JV 0610/VIIK1**	**Mexico**	**MG008436**	**MG008469**		**MH636396**	Du et al. (2020)	1. Annotated as *Fuscoporia* sp. in GenBank 2. Strain annotated as JV 0610/VII-Kout in the reference article
	JV 1109/482	USA	MG008439	MG008468	MN848799	MH636395	Du et al. (2020)	1. Annotated as *Fuscoporia* sp. in GenBank 2. Strain annotated as JV 1109/48 in the reference article
*F. monticola*	**Dai 11860**	**China**	**MG008406**	**MG008457**		**MH636390**	Du et al. (2020)	Annotated as *Fuscoporia* sp. in GenBank for ITS
*F. palomari*	JV 1305/3-J	USA	MN816738	MN810028		MN848801	Chen et al. (2020)	
*F. plumeriae*	Dai 17814	Singapore	MN816714	MN810011		MN848845	Chen et al. (2020)	Country annotated as USA in GenBank for ITS
	Dai 18820	Australia	MN816722	MN810012	MN848770	MN848844	Chen et al. (2020)	
	**Dai 18858**	**Australia**	**MN816712**	**MN810010**	**MN848769**	**MN848843**	Chen et al. (2020)	Country annotated as China in GenBank for ITS
*F. pulviniformis*	CMW45308	South Africa	MH599100	MH599124		MT108958	Tchoumi et al. (2020)	
	CMW47816	South Africa	MH599101	MH599125		MT108959	Tchoumi et al. (2020)	
	CMW48060	South Africa	MH599103	MH599126		MT108961	Tchoumi et al. (2020)	
	CMW48600	South Africa	MH599102	MH599127		MT108960	Tchoumi et al. (2020)	
	Dai 17255	China	MH050747	MH050761	MH079396		Chen and Dai (2019)	
*F. ramulicola*	**Dai 15723**	**China**	**MH050749**	**MH050762**	**MH079398**	**MN848824**	Chen and Dai (2019)	
	Dai 16155	China	MH050750	MH050763	MH079399	MN848825	Chen and Dai (2019)	
*F. reticulata*	SFC20121010-19	Republic of Korea	ON427766				This study	
	**SFC20160115-16**	**Republic of Korea**	**ON427761**	**ON427791**	**ON464727**	**ON479800**	**This study**	
*F. rhabarbarina*	Dai 16226	China	MN816743	MN810035	MN848784	MN848838	Chen et al. (2020)	Annotated as *Phellinus rhabarbarinus* in GenBank
	Dai 16550	China	MN816744	MN810036	MN848785	MN848836	Chen et al. (2020)	Annotated as *Phellinus rhabarbarinus* in GenBank
*F. roseocinerea*	JV 1109/78-J	USA	MN816742	MN810032		MN848820	Chen et al. (2020)	Strain annotated as JV 1109/78 in the reference article
	*JV 1407/84*	*Costa Rica*	*MN816740*	*MN810030*		*MN848819*	Chen et al. (2020)	
*F. semicephala*	**SFC20170524-08**	**Republic of Korea**	**ON427764**	**ON427794**	**ON464730**	**ON479803**	**This study**	
	SFC20170712-20	Republic of Korea	ON427787	ON427815		ON479819	This study	
*F. senex*	Dai 15775	China	MN816746	MN810038	MN848787	MN848834	Chen et al. (2020)	
	Dai 17043	China	MN816747	MN810039	MN848786	MN848835	Chen et al. (2020)	
	Dai 17132	China	MN816745	MN810037	MN848783	MN848833	Chen et al. (2020)	Specimen information not found in the reference article
*F. septiseta*	**Dai 12820**	**USA**	**MG008405**	**MN810033**		**MH636394**	Chen et al. (2019)	Annotated as *Fuscoporia* sp. in GenBank for ITS
*F. setifera*	Dai 15706	China	MH050759	MH050769	MN159391	MN848842	Chen and Dai (2019)	
	Dai 15710	China	MH050758	MH050767	MN159390	MN848841	Chen and Dai (2019)	
*F. shoreae*	Dai 17800	Singapore	MN816733	MN810024		MN848814	Chen et al. (2020)	Annotated as *Fuscoporia* sp. in GenBank
	Dai 17806	Singapore	MN816734	MN810025		MN848815	Chen et al. (2020)	Annotated as *Fuscoporia* sp. in GenBank
	**Dai 17818**	**Singapore**	**MN816735**	**MN810026**		**MN848816**	Chen et al. (2020)	Annotated as *Fuscoporia* sp. in GenBank
*F. sinica*	**Dai 15468**	**China**	**MG008412**	**MG008459**		**MH636392**	Chen et al. (2019)	Annotated as *Fuscoporia* sp. in GenBank for ITS and nrLSU
	Dai 15489	China	MG008407	MG008458	MN848798	MH636393	Chen et al. (2019)	Annotated as *Fuscoporia* sp. in GenBank for ITS and nrLSU
*F. subchrysea*	**Dai 16201**	**China**	**MN816708**	**MN809997**	**MN848796**	**MN848811**	Chen et al. (2020)	Country annotated as Thailand in GenBank for ITS
	Dai 17656	China	MN816709	MN809998		MN848812	Chen et al. (2020)	Country annotated as Thailand in GenBank for ITS
*F. subferrea*	Dai 16326	China	KX961097	KY053472	MH079400	MN848826	Chen and Dai (2019)	
	**Dai 16327**	**China**	**KX961098**	**KY053473**	**MH079401**		Chen and Dai (2019)	
*F. torulosa*	Dai 15518	China	MN816732	MN810023	MN848781	MN848827	Chen et al. (2020)	
	JV 1312/19-Kout	Spain	KX961107	KY189107		MH636406	Chen and Dai (2019)	
	JV 1405/2	Czech Republic	KX961106	KY189106		MH636405	Chen and Dai (2019)	
*F. viticola*	He 2081	USA	MN121829	MN121770			Chen and Dai (2019)	
	He 2123	USA	MN816725	MN810017			Chen et al. (2020)	
*F. yunnanensis*	*Cui 8182*	*China*	*MH050756*	*MN810029*	*MN848789*		Chen and Dai (2019)	

Type-derived sequences are in bold, and type locality-derived sequences are indicated by an italic

Maximum Likelihood (ML) and Bayesian inference (BI) trees were constructed using concatenated sequences. A nucleotide substitution model for each genetic marker was estimated and employed by respective phylogeny tools on the CIPRES Science Gateway Web server—the ML tree was inferred using RAxML v.8.2.12 (Stamatakis 2014) with 1000 replications, and the BI tree was constructed with ExaBayes v.1.5.1 (Aberer et al. 2014), starting from random trees. BI trees were sampled every 500th generation from one million generations. A 75% majority rule consensus tree was constructed after removing the first 5% of the trees, and the Bayesian Posterior Probabilities (BPP) were calculated from the remaining trees. The outgroup sequences included in the analyses were *Phellinidium fragrans* (CBS 202.90) and *Phellinidium ferrugineofuscum* (Cui 10042).

### Morphological study

Macro-morphological characters, including hymenophore type, tube length, pore size, and color of the trama, tube, and subiculum, were analyzed for all the studied specimens. Observations were performed using a Nikon SMZ1500 stereomicroscope (Nikon, Japan) at 7.5–60 × . All measured colors followed the *Methuen Handbook of Colours* (Kornerup and Wanscher 1978).

To examine the micromorphological characters, the hymenophore tissue of the new species was cut and mounted in 5% KOH. Features, including the size and shape of basidia, basidiospores, cystidia, hyphae, and setae were observed under a Nikon 80i compound light microscope (Nikon, Tokyo) at 100 × to 400 × magnification. The setae observed were classified into two categories: ‘hymenial setae’ for setae occurring in the hymenium, and ‘mycelial setae’ for the long setae present in the subiculum or trama (Chen et al. 2019). For the description of hyphae, the lumen was described as ‘wide’ if it was wider than the wall of the hypha, ‘medium’ if of similar width, and ‘narrow’ for narrower width.

For measurements, 20–60 elements were selected for each specimen. For basidiospores, 5% of the extreme values from each end were excluded and are given in parentheses. ‘L’ refers to the mean basidiospore length, ‘W’ to the mean basidiospore width, and ‘Q’ to the average length: width ratio of the basidiospores. ‘*n* = x/y’ refers to the number of basidiospores measured (x) and the number of specimens (y). Cyanophilic and iodine reactions of basidiospores were tested using Cotton Blue and Melzer’s reagent. ‘CB–’ refers to acyanophilous, and ‘IKI–’ indicates neither amyloid nor dextrinoid.

## RESULTS

### ITS phylogenetic analysis

**Fig. 1:**
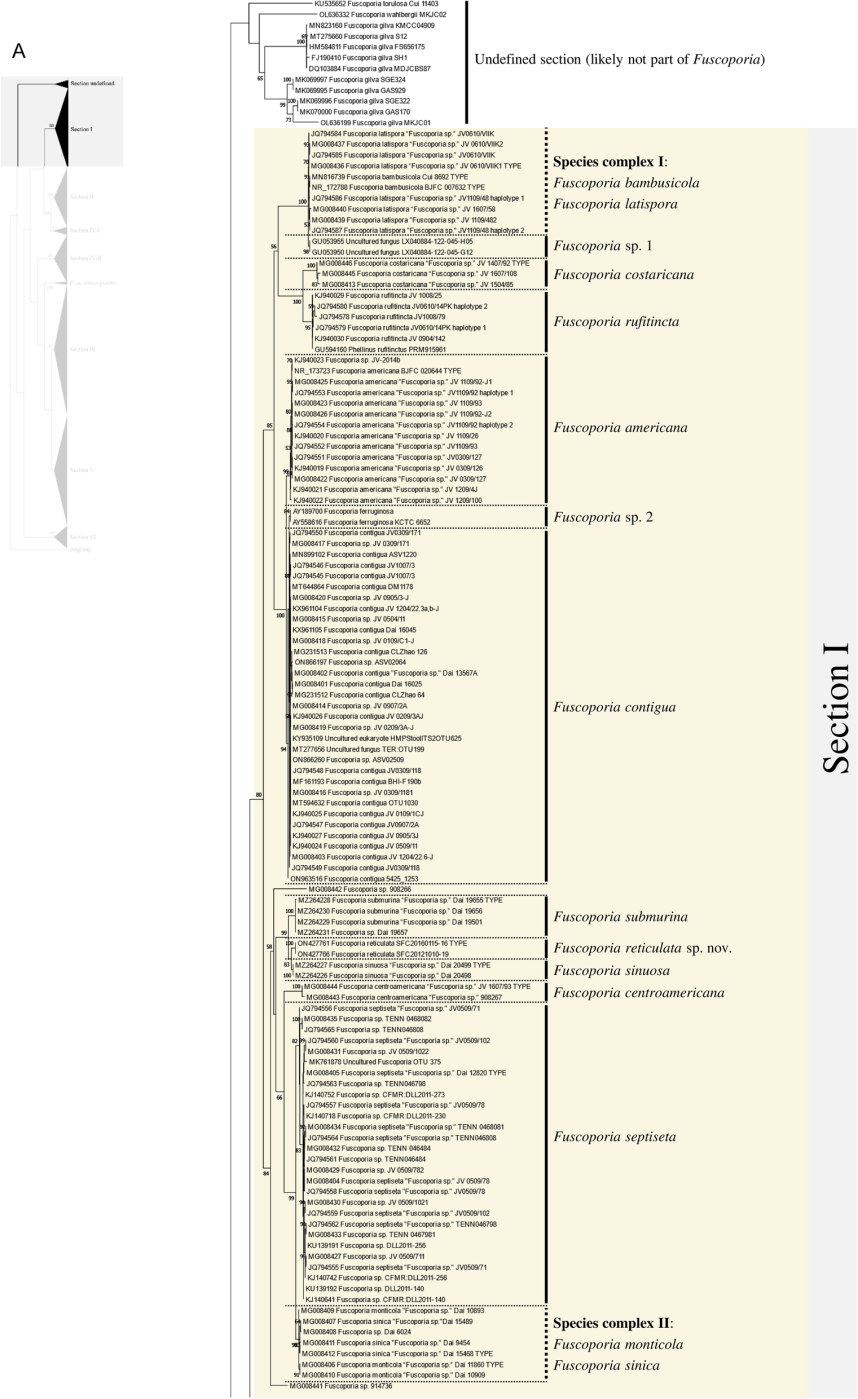

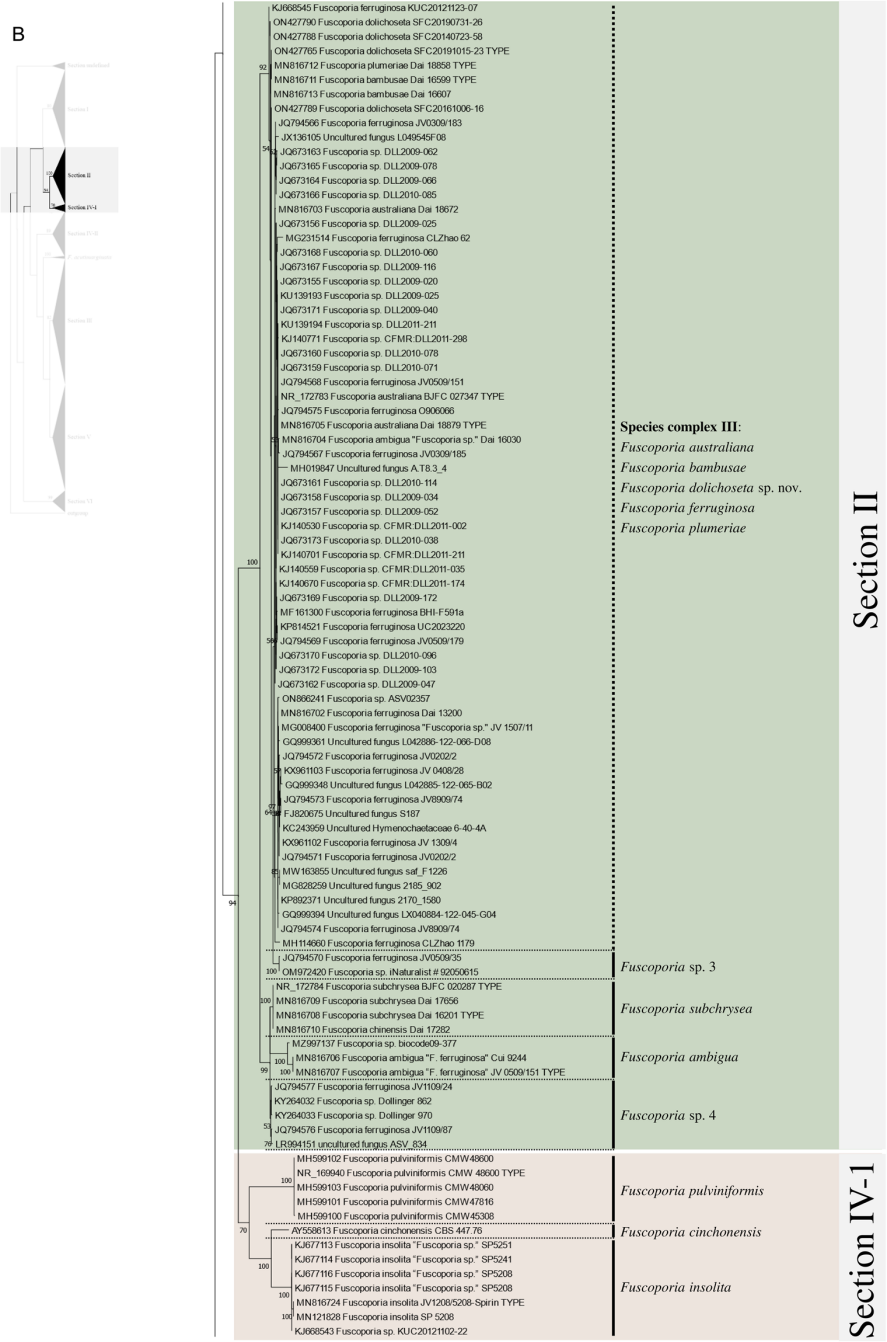

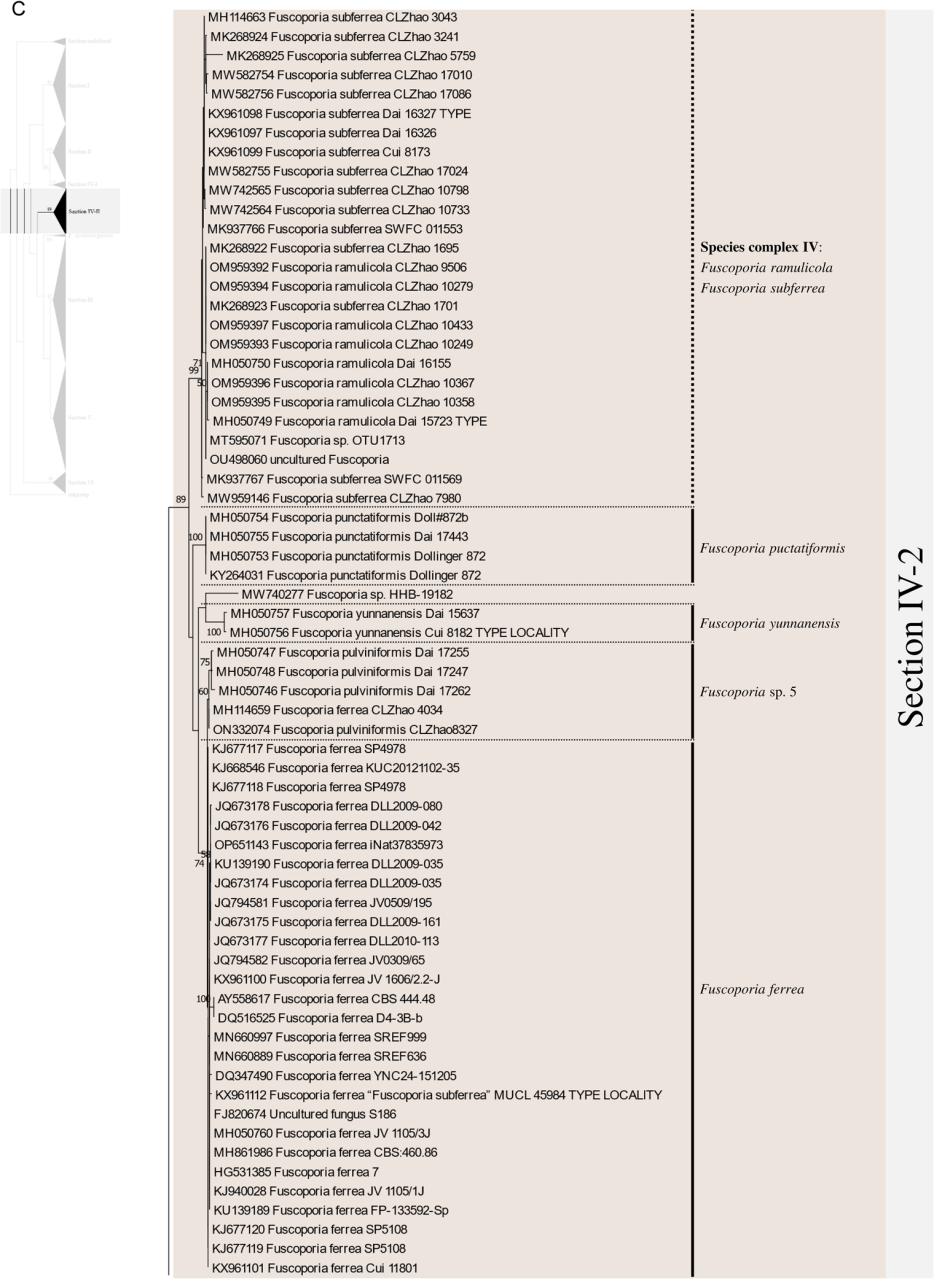

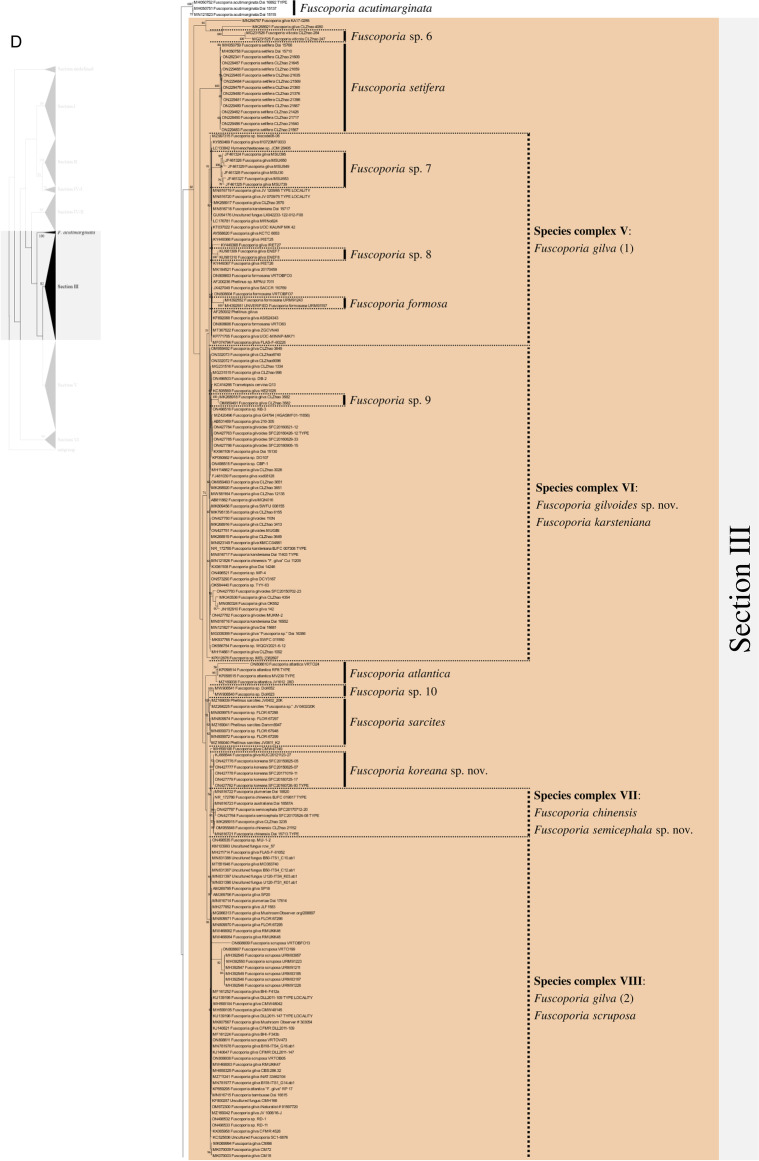

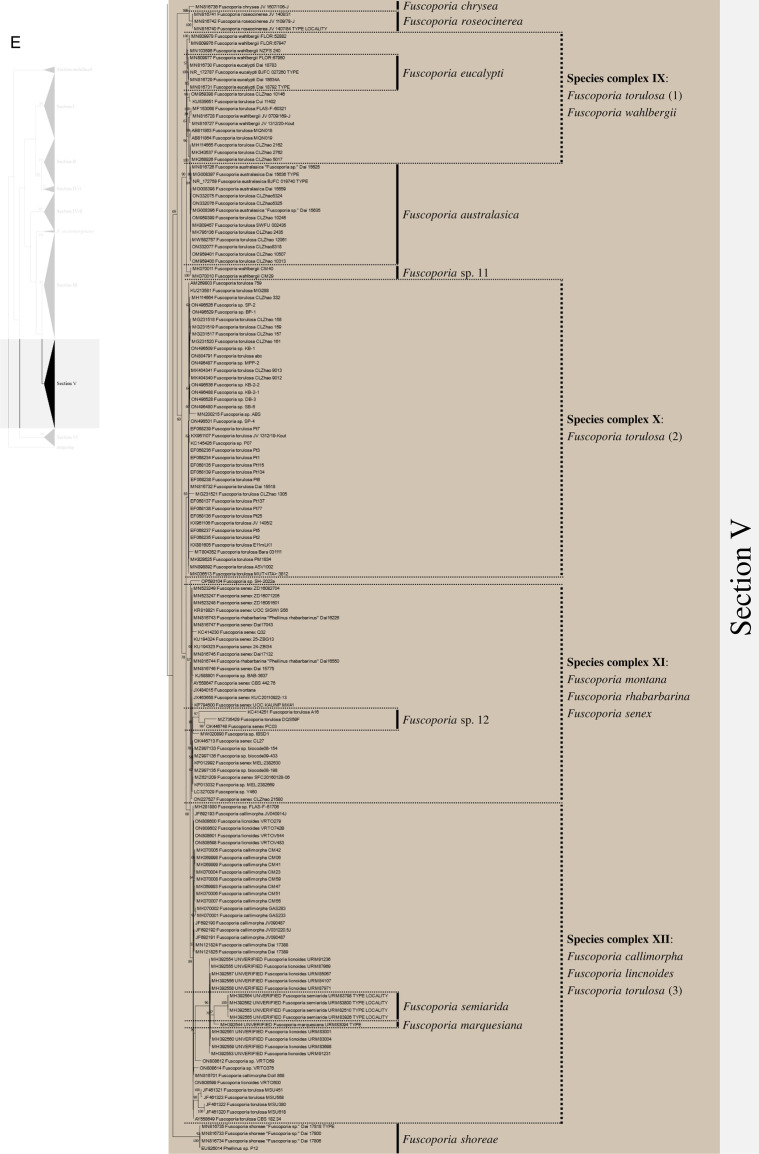

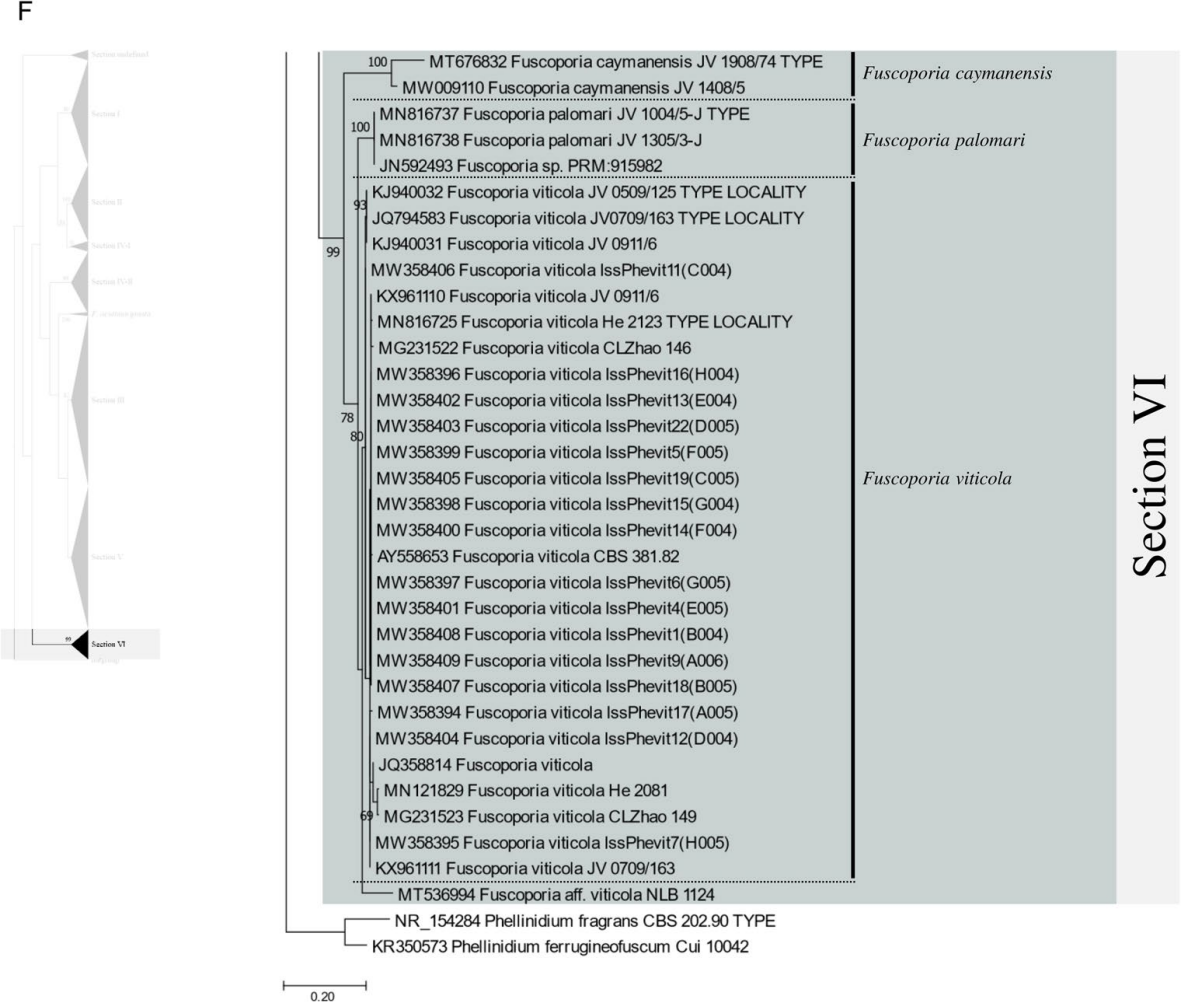
ML tree of *Fuscoporia* ITS sequences by sections. **A** undefined section and section I, **B** section II and IV-1, **C** section IV-2, **D** section III, **E** section V, **F** section VI. Simple display of sections assigned based on multi-marker phylogenetic analyses is shown on the left. Bootstrap values over 50% are indicated. Vertical dotted lines indicate species complexes, and singleton sequences are left unlabelled

Excluding the undefined Sect. (12 sequences), 658 ITS sequences were assessed within the genus *Fuscoporia* with 42 type-derived sequences (34 species) and 14 type locality sequences (seven species). The phylogenetic tree based on 1710 nucleotide bases (including gaps) of the ITS region contained clades of species that mostly corresponded to the six sections in Chen et al. (2020) (Fig. 1). The order of the sections in the ITS tree followed the order of the multi-marker phylogenetic tree described below. Unspecified sequences, such as ‘*Fuscoporia* sp.,’ ‘*Hymenochaetales* sp.,’ and ‘Uncultured fungus’ from GenBank accounted for 196 (29.8%) of all sequences. Some sequences with confident species identities in published articles, including a few type-derived sequences, were annotated as ‘*Fuscoporia* sp.’ in GenBank. Only 353/658 (53.6%) were correctly annotated if synonyms were accounted for. The misidentified sequences included MN816710 (*F. chinensis*) in the *F. subchrysea* clade (Section II) and *F. torulosa* sequences in the *F. australasica* clade (Section V). Some sequences were annotated differently across multiple studies. For example, *F. australasica* sequences (GenBank accession no. MG008397 and MG008398) were annotated as either *F. australasica* or *F. wahlbergii* in three different research articles (Additional file 3: Table S2).

Based on the re-identification of all sequences with respect to the type- or type locality-derived sequences and reliable sequences, the mislabelled sequences were annotated in the phylogenetic tree by species clades to reflect their true identities (Fig. 1). Unspecified sequences included potentially new species that have not yet been described (*Fuscoporia* sp. 1 to 12). All sequence validations and re-identifications are presented in Additional file 3: Table S2. The top BLAST hits for each GenBank accession are listed to assess the accuracy of BLAST-based species identification. For 11.7% (76/649, excluding the newly generated accessions in this study), the topmost hit was different from the true identity of the query sequence, and for 32.0% (208/649), the BLAST result did not have a specified identity to the species level (Additional file 3: Table S2).

Regrettably, not all sequences were confidently re-identified, as an ITS analysis has a low resolution of species delimitation in some groups. This resulted in 12 species complexes across the six sections. Seven species complexes each formed a monophyletic clade with type-derived or reliable sequences for two or more species (Fig. 1): *F. bambusicola*/*F. latispora* (Complex I) and *F. monticola*/*F. sinica* (Complex II) in section I, *F. australiana*/*F. bambusae*/*F. dolichoseta* sp. nov./*F. ferruginosa*/*F. plumeriae* (Complex III) in section II, *F. ramulicola*/*F. subferrea* (Complex IV) in section IV-2, *F. gilvoides* sp. nov./*F. karsteniana* (Complex VI) and *F. chinensis*/*F. semicephala* sp. nov. (Complex VII) in section III, and *F. montana*/*F. rhabarbarina*/*F. senex* (Complex XI) in section V. In contrast, five species complexes (Complexes V, VIII, IX, X, and XII) consisted of sequences in paraphyletic clades for species *F. gilva* and *F. torulosa*. These species complexes did not have any type-derived sequences to determine which monophyletic clade corresponded to their respective species. This prevented us from determining which clade consisted of misidentified sequences.

### Multi-marker phylogenetic analyses

**Fig. 2:**
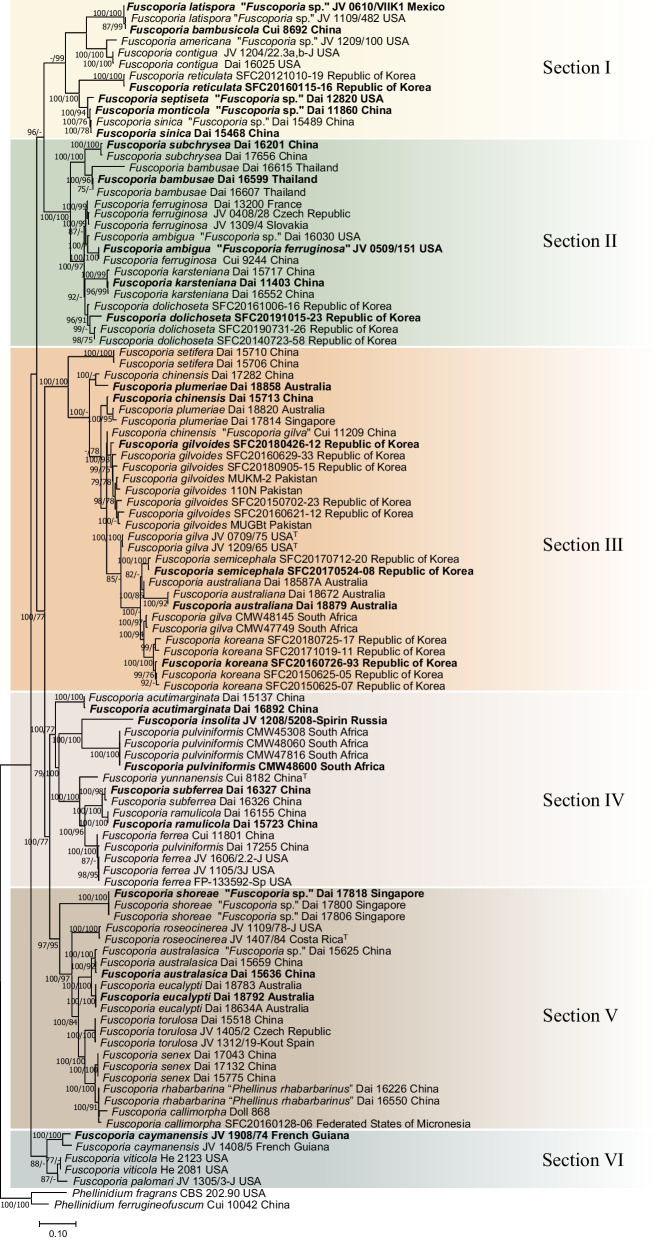
*Fuscoporia* phylogeny inferred using BI and ML methods based on concatenated ITS + nrLSU + *rpb2* + *tef1* sequences. Tree topology is from BI analysis, and statistical values (BI/ML) above 75% are designated at each node out of 100. Type-derived sequences are in bold and labels from GenBank are given in quotation marks. Superscript ‘^T^’ indicates type locality-derived sequences

The references in the ITS + nrLSU + *rpb2* + *tef1* multi-marker dataset comprised 82 strains from 35 species in *Fuscoporia* (Table 1). The concatenated multiple sequence alignment was 3286 bases long, including gaps. The ITS1 region comprised 301 bases, 5.8S of 157 bases, ITS2 region of 285 bases, nrLSU of 1379 bases, *rpb2* of 619 bases, and *tef1* of 545 bases (exon 1: 135 bases, intron 1: 71 bases, exon 2: 137 bases, intron 2: 63 bases, and exon 3: 139 bases), including gaps (Additional file 5: Data S2). Clades were divided into six sections (I to VI) following the division of Chen et al. (2020). The “Undefined section” did not belong to any of the six sections and was removed from the analysis (Fig. 1). Multi-marker phylogenetic analyses resolved five species complexes from the ITS tree (Fig. 2): *F. australiana*/*F. bambusae*/*F. dolichoseta* sp. nov. (Complex III, partial), *F. ramulicola*/*F. subferrea* (Complex IV), *F. gilvoides* sp. nov./*F. karsteniana* (Complex VI), *F. chinensis*/*F. semicephala* sp. nov. (Complex VII), and *F. rhabarbarina*/*F. senex* (Complex XI, partial). *Fuscoporia plumeriae* in complex III formed a new species complex with *F. chinensis* in complex VII. Species complexes I and II remained monophyletic, possibly because of low genetic divergence between the two species within each clade or the limited number of specimens available for analysis.

Apart from the aforementioned issues of ambiguously labelled or misidentified GenBank sequences, there were additional issues. The main issue was the conflicting data annotations between GenBank records and the corresponding research articles, which is more problematic for multi-marker analyses. Sequences from published papers for species such as *F. americana*, *F. shoreae*, and *F. sinica* were labelled as "*Fuscoporia* sp." in GenBank, including some type-derived sequences (Table 1). There was also a mislabelled type-derived sequence, *F. ambigua* (JV 0509/151), which was annotated as *F. ferruginosa* in GenBank. Additionally, some GenBank sequences were labelled with different specimen names. For example, "JV 1208/5208-Spirin" *F. insolita* specimen was recorded as Spirin 5208 in the reference article (Chen et al. 2020). Mismatches were also observed in the annotated features between the genetic markers from a single specimen. Most had disagreeing country labels, as observed for *F. subchrysea* specimens Dai 16201 and Dai 17656. Both specimens were recorded to have originated from China in the research article, but the country annotated for the ITS sequences was Thailand. Specimens of *F. bambusae* (Dai 16599, Dai 16607, and Dai 16615), all from Thailand, were recorded to have originated from either China or the USA in GenBank. Other minor issues included the lack of information on specimens in the annotated published reference paper and disagreeing species identities among sequences of the same specimen.

### New species assessments

**Fig. 3:**
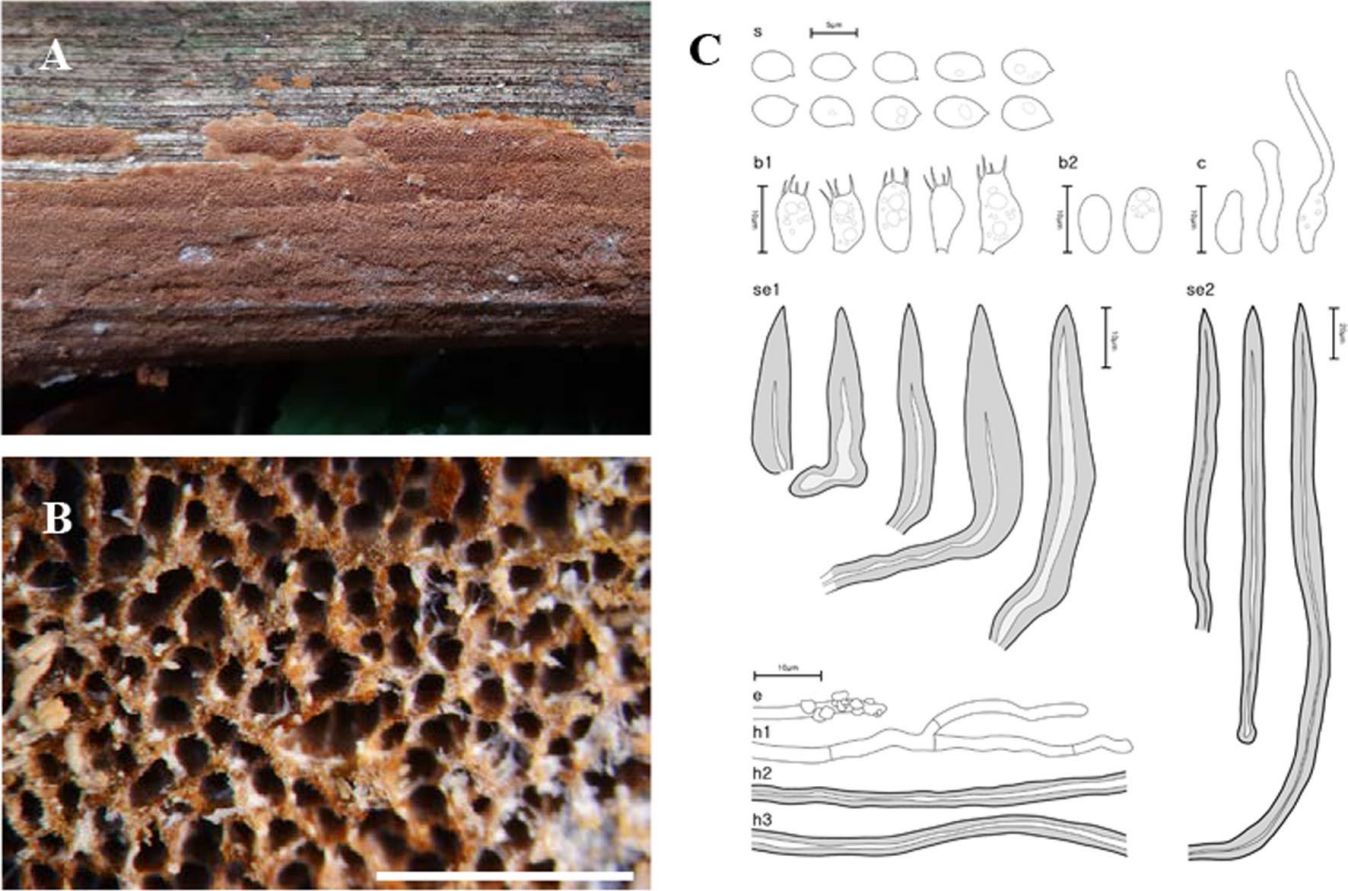
Morphological characters of *Fuscoporia dolichoseta* (SFC20191015-23, holotype). **A** basidiome, **B** pore surface, **C** drawings of microscopic features, where ‘s’ refers to basidiospores, ‘b1’ basidia, ‘b2’ basidioles, ‘c’ cystidioles, ‘se1’ hymenial setae, ‘se2’ mycelial setae, ‘e’ encrusted generative hyphae at dissepiment edge, ‘h1’ generative hyphae, ‘h2’ skeletal hyphae in trama, ‘h3’ skeletal hyphae in subiculum. Scale bar for the pore surface is 1 mm

**Fig. 4:**
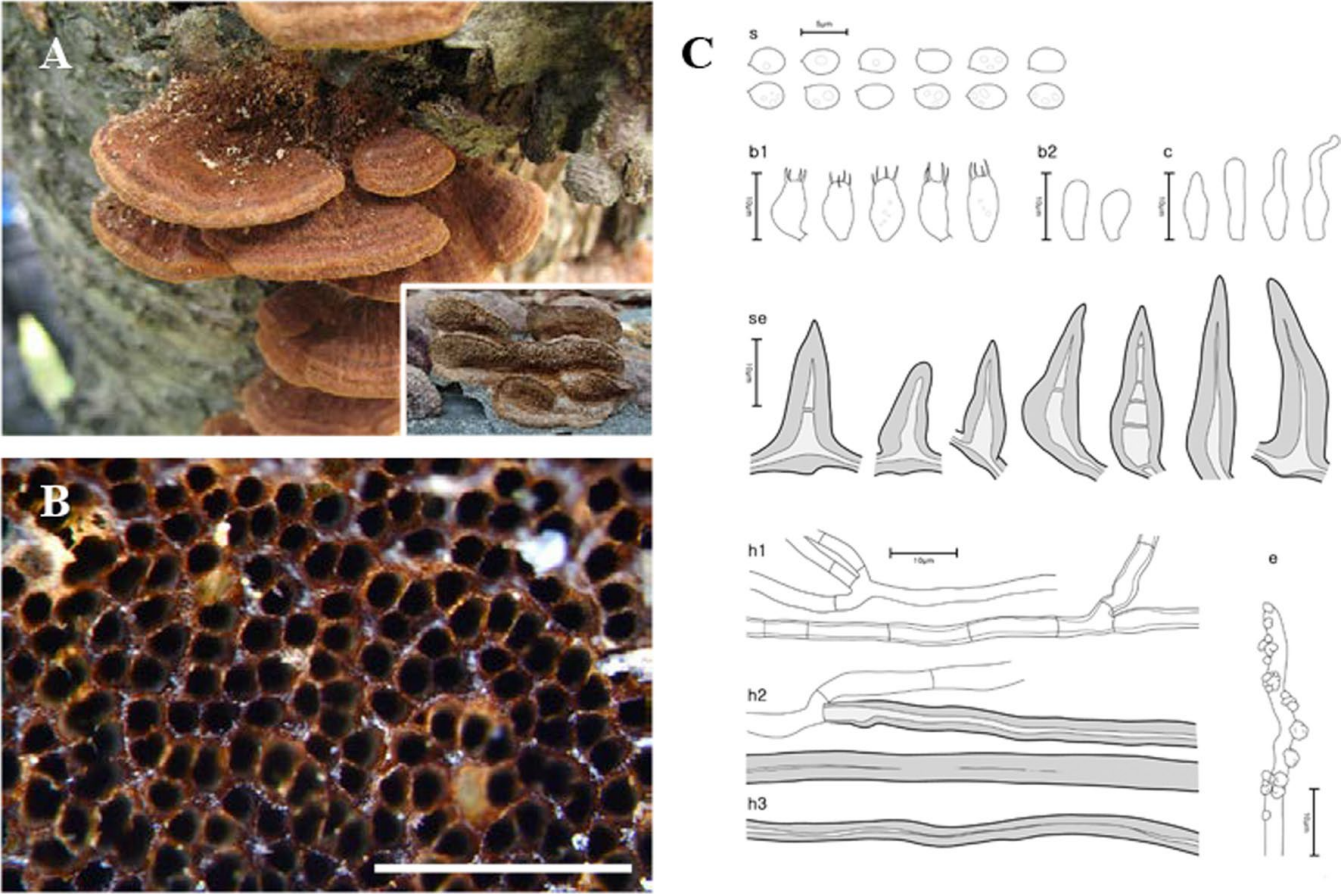
Morphological characters of *Fuscoporia gilvoides* (SFC20180426-12, holotype). **A** basidiome, **B** pore surface, **C** drawings of microscopic features, where ‘s’ refers to basidiospores, ‘b1’ basidia, ‘b2’ basidioles, ‘c’ cystidioles, ‘se’ hymenial setae, ‘h1’ generative hyphae, ‘h2’ skeletal hyphae in trama, ‘h3’ skeletal hyphae in context, ‘e’ encrusted generative hyphae at dissepiment edge. Scale bar for the pore surface is 1 mm

**Fig. 5:**
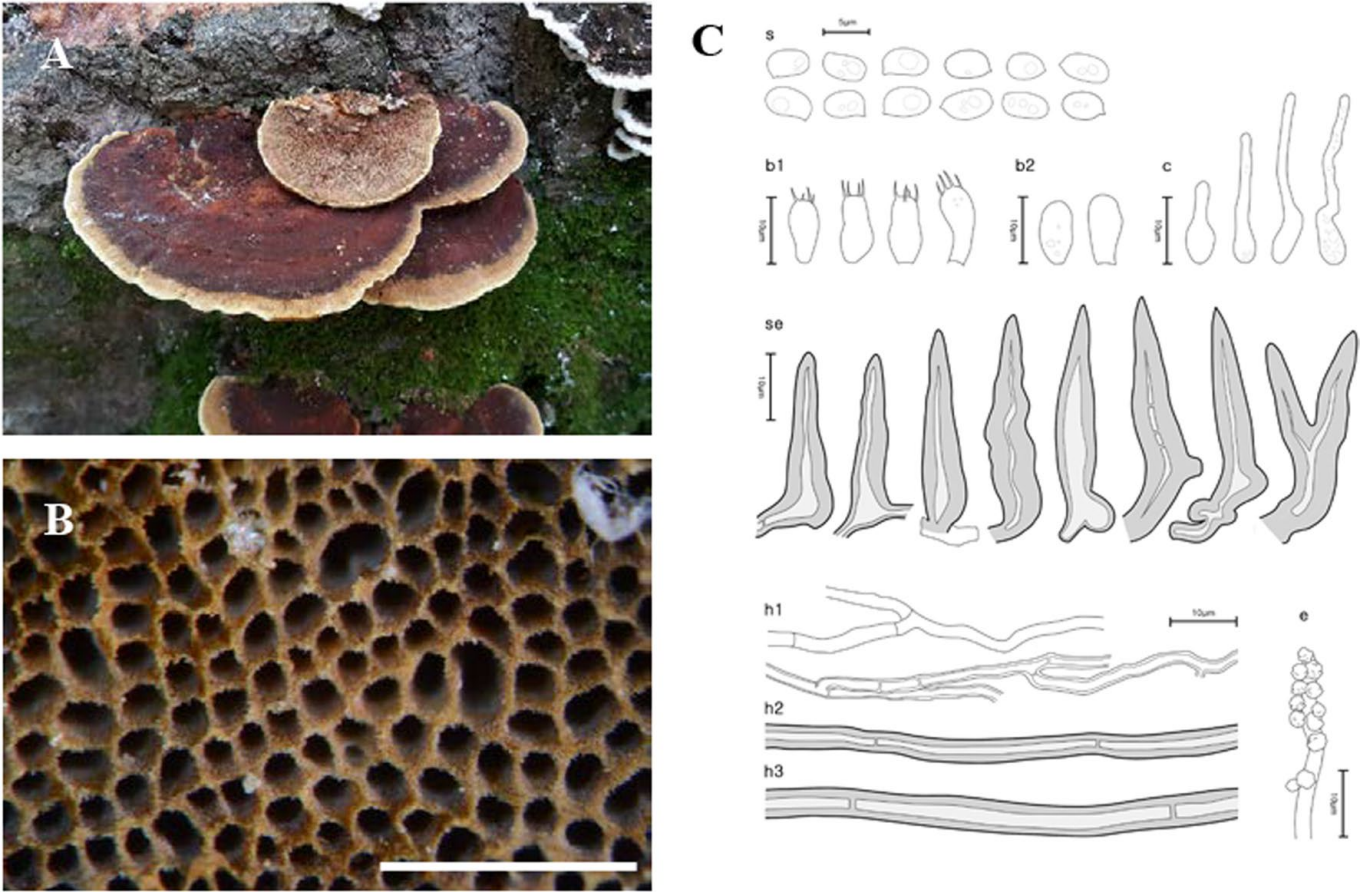
Morphological characters of *Fuscoporia koreana* (SFC20160726-93, holotype). **A** basidiome, **B** pore surface, **C** drawings of microscopic features, where ‘s’ refers to basidiospores, ‘b1’ basidia, ‘b2’ basidioles, ‘c’ cystidioles, ‘se’ hymenial setae, ‘h1’ generative hyphae, ‘h2’ skeletal hyphae in trama, ‘h3’ skeletal hyphae in context, ‘e’ encrusted generative hyphae at dissepiment edge. Scale bar for the pore surface is 1 mm

**Fig. 6:**
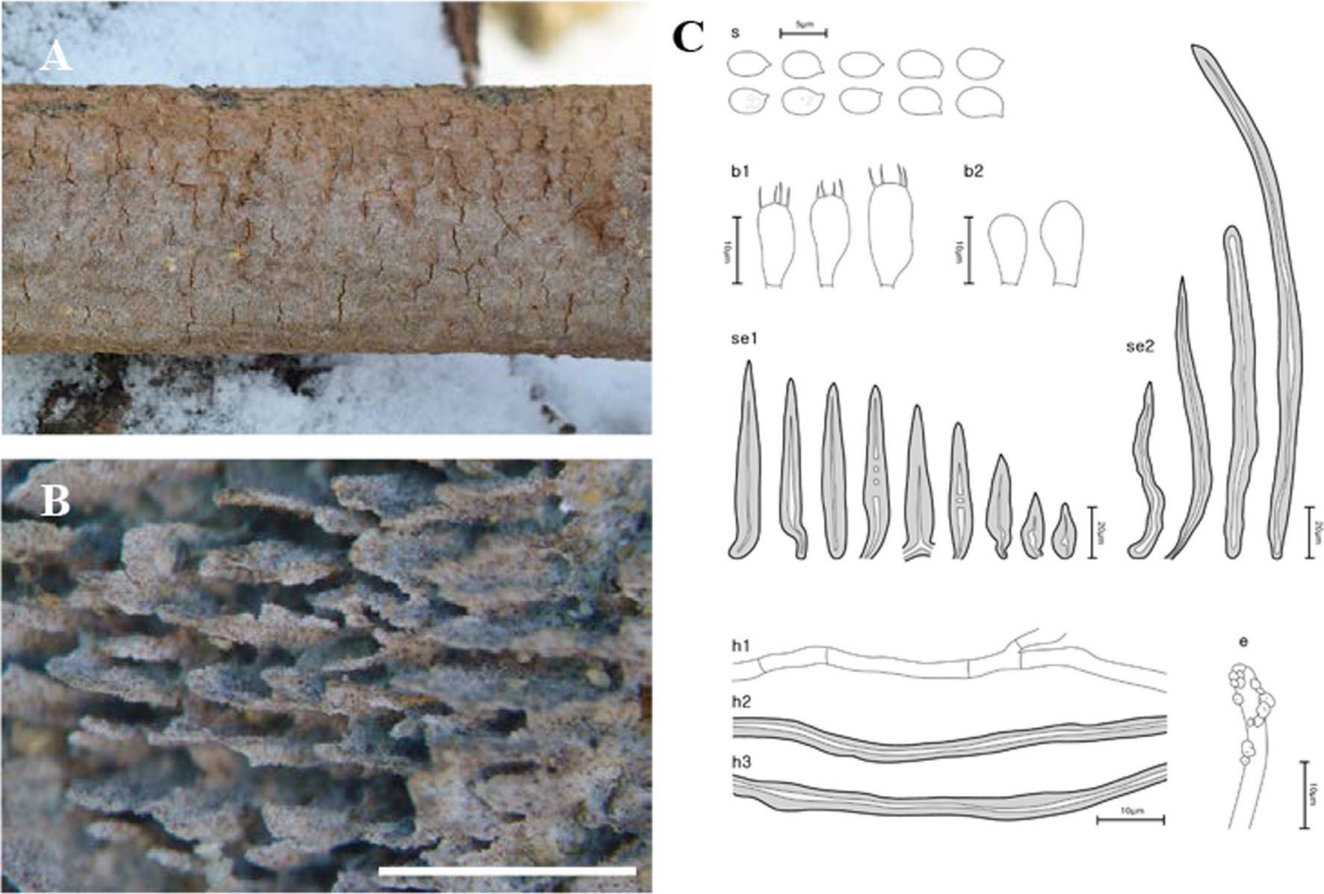
Morphological characters of *Fuscoporia reticulata* (SFC20160115-16, holotype). **A** basidiome, **B** pore surface, **C** drawings of microscopic features, where ‘s’ refers to basidiospores, ‘b1’ basidia, ‘b2’ basidioles, ‘se1’ hymenial setae, ‘se2’ mycelial setae, ‘h1’ generative hyphae, ‘h2’ skeletal hyphae in trama, ‘h3’ skeletal hyphae in subiculum, ‘e’ encrusted generative hyphae at dissepiment edge. Scale bar for the pore surface is 1 mm

**Fig. 7:**
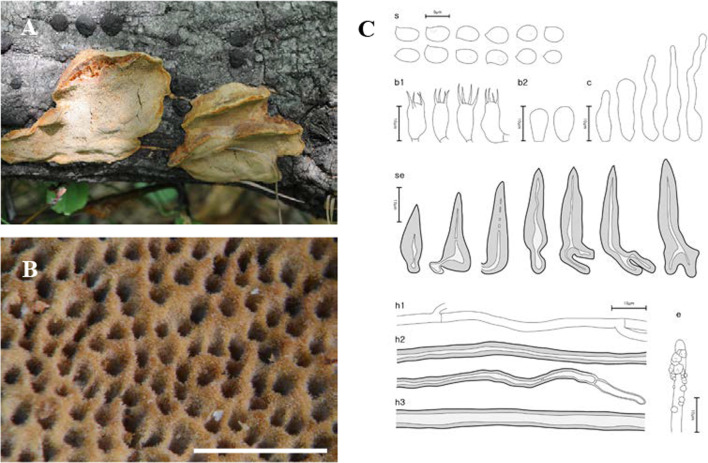
Morphological characters of *Fuscoporia semicephala* (SFC20170524-08, holotype). **A** basidiome, **B** pore surface, **C** drawings of microscopic features, where ‘s’ refers to basidiospores, ‘b1’ basidia, ‘b2’ basidioles, ‘c’ cystidioles, ‘se’ hymenial setae, ‘h1’ generative hyphae, ‘h2’ skeletal hyphae in trama, ‘h3’ skeletal hyphae in context, ‘e’ encrusted generative hyphae at dissepiment edge. Scale bar for the pore surface is 1 mm

The 21 newly analyzed specimens in this study formed five well-supported monophyletic clades in both BI and ML analyses (Fig. 2). The new *Fuscoporia* clade with the type specimen SFC20191015-23 is described as *Fuscoporia dolichoseta* sp. nov. (Bootstrap BI = 96/ML = 91). The clade with the type specimen SFC20180426-12 was designated as *Fuscoporia gilvoides* sp. nov. (100/98), SFC20160726-93 (type) as *Fuscoporia koreana* sp. nov. (100/100), SFC20160115-16 (type) as *Fuscoporia reticulata* sp. nov. (100/100), and SFC20170524-08 (type) as *Fuscoporia semicephala* sp. nov. (100/100). The five new species exhibited prominent morphological differences that distinguish them from other *Fuscoporia* species. The basidiome, pore surface, and microscopic features of the new species are presented in Figs. 3, 4, 5, 6, and 7, respectively.

## TAXONOMY

***Fuscoporia dolichoseta*** Y. Cho, D. Kim & Y. W. Lim, **sp. nov**. (Fig. 3).

MycoBank: MB 844763.

*Etymology*: ‘dolichoseta’ describes the long and narrow setae of the species.

*Type*: **Republic of Korea**: Gangwon-do, Taebaek-si, Sodo-dong, 37°07′07.0 ʹʹ N 128°57′02.0ʹʹ E, 839 m, Mt. Taebaek, mixed forest, on dead angiosperm trunk, 15 Oct 2019, *Young Woon Lim* (SFC20191015-23—holotype).

*Diagnosis*: Basidiomes perennial, resupinate, tuberculate, develop in temperate regions; pores irregular; mycelial setae abundant, dark brown, aseptate, 52.0–266.1 × 6.6–12.8 μm; cystidioles fusoid, lageniform or cylindric-flexuous; basidiospores ellipsoid to ovoid, smooth, some with 1–3 guttules, 4.4–5.3 × 3.1–3.7 μm.

*Description*: *Basidiome* perennial, resupinate, tuberculate, inseparable up to 1 mm thick at center. *Pore surface* dark brown (9F7), uncracked when dry, margin sterile, 1–4 mm wide, beige (5B4), paler than pore surface. *Pores* more or less round, sinuous or irregular, 6–7 pores per mm. *Tubes* pale grey (20B1), corky, to 0.9 mm deep, dissepiments to 0.8 mm thick, entire, abundant hymenial setae seen under stereomicroscope. *Subiculum* light yellow (4A4), corky, to 1.8 mm thick.

*Hyphal system* dimitic; generative hyphae hyaline to pale yellow, thin-walled, branched, simple septate, 1.7–2.9 μm wide in tube, 1.8–2.3 μm wide in subiculum, some at dissepiment edge encrusted with crystals; skeletal hyphae dominant in both tubes and subiculum, more loose in subiculum, rusty brown to golden yellow, thick-walled with medium to narrow lumen, unbranched, 2–3 secondary septa present at the apex, 2.2–4.3 μm wide in tube, 2.1–2.7 μm wide in subiculum.

*Basidia* barrel-shaped to utriform, four sterigmata, simple septum at the base, mostly guttulate, 8.6–12.3 × 5.0–6.3 μm; basidioles smaller in size compared to basidia. *Basidiospores* ellipsoid to ovoid, hyaline, thin-walled, smooth, some with 1–3 guttules, IKI–, CB–, (3.9–)4.4–5.3(–5.6) × (2.7–)3.1–3.7(–4.0) μm, L = 4.83 μm, W = 3.37 μm, Q = 1.44 (*n* = 60/2). *Cystidioles* fusoid, lageniform or cylindric-flexuous, hyaline, thin-walled, 8.5–33.4 × 2.9–5.5 μm. *Hymenial setae* subulate, acute at the apex, some with bent and elongated base, dark brown, thick-walled, aseptate, 21.6–82.5 × 4.7–10.8 μm. *Mycelial setae* abundant in subiculum, straight, acute at the apex, dark brown, thick-walled, aseptate, 52.0–266.1 × 6.6–12.8 μm.

*Ecology/Substrate/Host*: On dead trunks and branches of angiosperms in temperate forests, causing a white rot, to *ca*. 920 m above sea level in altitude.

*Distribution*: Republic of Korea.

*Additional specimens examined***:** See Additional file 6: Data S3.

*Notes*: *Fuscoporia dolichoseta* is phylogenetically close to *F. ambigua* and *F. ferruginosa*. *Fuscoporia ambigua* may be differentiated by annual basidiomes and larger basidia of 14–18 × 4.5–6.0 μm (Du et al. 2020). Similarly, *F. ferruginosa* may also be distinguished by larger basidia of 11–14 × 4.5–6.5 μm and basidiospores of 5.0–6.5 × 3.0–3.5 μm (Núñez and Ryvarden 2000). *Fuscoporia dolichoseta* is also closely related to *F. karsteniana*. *Fuscoporia karsteniana* may be differentiated by the absence of cystidioles and larger basidia of 14–16 × 4–6 μm (Chen et al. 2020).

***Fuscoporia gilvoides*** Y. Cho, D. Kim & Y. W. Lim, **sp. nov**. (Fig. 4).

MycoBank: MB 844764.

*Etymology*: ‘gilvoides’ indicates the species’ similarity to *F. gilva*.

*Type*: **Republic of Korea**: Gangwon-do, Gangneung-si, Seongsan-myeon, 37°42′35.0ʹʹ N 128°47′03.0 ʹʹ E, 417 m, Daegwallyeong Natural Recreation Forest, mixed forest, on *Carpinus laxiflora,* 26 Apr 2018, *Hyun Lee*, *Min-Ji Kim*, & *Abel Severin Lupala* (SFC20180426-12—holotype).

*Diagnosis*: Basidiomes perennial, effused-reflexed or pileate, found in Asia; pores 6–7 per mm; hymenial setae abundant, often bi-radicated, some septate, 17.7–33.7 × 5.5–9.4 μm; cystidioles fusoid, lageniform or cylindric; basidiospores ellipsoid to ovoid, guttulate, 3.6–4.1 × 2.3–2.8 μm.

*Description*: *Basidiome* perennial, pileate, sometimes effused-reflexed, solitary to imbricate. *Pileus* dimidiate, undulate, to 2.2 cm in diam, 1.3 cm thick at center. *Pileal surface* concentrically sulcate and zonate, nodulose, rugose, sometimes velutinate, pale beige brown (5B3); margin obtuse to slightly acute, pale brown (6D4), to 2 mm. *Pore surface* azukiiro (9F8), margin sterile, to 1 mm wide, caramel (7D8), paler than pore surface. *Pores* more or less circular, 6–7 pores per mm. *Tubes* grey (22C1), corky, to 3.6 cm deep, dissepiments to 0.1 mm thick, entire, abundant hymenial setae seen under stereomicroscope. *Context* dark beige (3C4) to pale brown (7D5), corky, to 4.0 mm thick.

*Hyphal system* dimitic; generative hyphae hyaline to pale yellow, thin- to slightly thick-walled, branched, simple septate, 1.8–3.8 μm wide in tube, 2.2–3.7 μm wide and rare in context, some at dissepiment edge coarsely encrusted with crystals; skeletal hyphae dominant in both context and tube, rusty brown to golden brown, thick-walled with narrow to solid lumen, unbranched, 2–3 secondary septa present at the apex, 2.5–5.2 μm wide in tube, 2.7–4.0 μm wide in context.

*Basidia* clavate to utriform, four sterigmata, simple septum at the base, 6.8–10.3 × 3.8–5.3 μm; basidioles shorter in length and width compared to basidia. *Basidiospores* ellipsoid to ovoid, hyaline, thin-walled, smooth, guttulate, IKI–, CB–, (3.3–)3.6–4.1(–4.3) × (2.1–)2.3–2.8(–3.3) μm, L = 3.85 μm, W = 2.55 μm, Q = 1.51 (*n* = 90/3). *Cystidioles* fusoid, lageniform or cylindric, hyaline, thin-walled, 9.7–17.4 × 3.0–4.3 μm. *Hymenial setae* frequent, subulate to ventricose, acute to acuminate at the apex, often bi-radicated, dark brown, thick-walled, some septate, 17.7–33.7 × 5.5–9.4 μm.

*Ecology/Substrate/Host*: Causes a white rot on dead trunks of angiosperms, including *Carpinus laxiflora*, *Prunus*, and *Quercus* in temperate forests at a wide range of altitude.

*Distribution*: Republic of Korea and Pakistan.

*Additional specimens examined*: See Additional file 6: Data S3.

*Notes*: *Fuscoporia chinensis* is similar to *F. gilvoides* but has annual basidiomes and larger basidia of 10–14 × 4–6 μm (Chen et al. 2020). *Fuscoporia gilva* is also closely related to *F. gilvoides* but has septate skeletal hyphae and larger basidiospores of 4–5 × 3–3.5 μm (Dai 2010). *Fuscoporia koreana* and *F. semicephala*, also from the Republic of Korea, are phylogenetically closely related to *F. gilvoides* but both have larger basidiospores than *F. gilvoides*.

***Fuscoporia koreana*** Y. Cho, D. Kim & Y. W. Lim, **sp. nov**. (Fig. 5).

MycoBank: MB 844765.

*Etymology*: After the country origin of the species, the Republic of Korea.

*Type*: **Republic of Korea**: Gyeonggi-do, Goyang-si, Deokyang-gu, Yongdu-dong, 37°37′48.6ʹʹ N 126°53′35.8ʹʹ E, 51 m, Royal Tombs, on dead angiosperm trunk, 26 Jul 2016, *Hyun Lee*, *Hae Jin Cho*, *Vladimir Li*, & *Ki Hyeong Park* (SFC20160726-93—holotype).

*Diagnosis*: Basidiomes pileate or effused-reflexed, found in temperate regions; pores 6–7 per mm; hymenial setae often bi-radicated, occasionally septate, 20.0–46.2 × 4.5–9.2 μm; basidiospores oblong-ellipsoid, few ovoid, guttulate, 3.9–4.8 × 2.3–2.7 μm.

*Description*: *Basidiome* perennial, pileate, sometimes effused-reflexed, solitary to imbricate. *Pileus* applanate, undulate, to 5.5 cm in diam., 1.1 cm thick at center. *Pileal surface* concentrically zonate and sulcate, scabrate, nodulose, azukiiro (10F6), pale yellow (1A2) in margin for up to 0.9 mm. *Pore surface* dark brown (8F7) in center, caramel (5C5) in margin for up to 0.7 mm. *Pores* circular, 6–7 pores per mm. *Tubes* pale grey (5C1), corky, to 6 mm deep, dissepiments projecting, entire, greyish yellow (3C3), to 0.1 mm thick, abundant hymenial setae seen under stereomicroscope. *Context* light yellow brown (4B5) to clay brown (5C7) near the tube, corky, to 5.5 mm thick.

*Hyphal system* dimitic; generative hyphae hyaline to greyish orange, thin- to slightly thick-walled, branched, simple septate, 1.5–3.2 μm wide in tube, 2.4–3.8 μm wide and rare in context, some at dissepiment edge coarsely encrusted with stellate crystals; skeletal hyphae dominant in both context and tube, rusty brown to caramel brown, thick-walled with medium to wide lumen, unbranched, frequently with septa, especially in context. 2.2–4.0 μm wide in tube, 3.2–5.5 μm wide in context.

*Basidia* clavate to utriform, hyaline, four sterigmata, simple septum at the base, 8.6–11.6 × 4.1–5.1 μm; basidioles shorter in length and of similar width as the basidia. *Basidiospores* oblong-ellipsoid, few ovoid, hyaline, thin-walled, smooth, guttulate, IKI–, CB–, (3.4–)3.9–4.8(–5.1) × (2.2–)2.3–2.7(–3.0) μm, L = 4.37 μm, W = 2.51 μm, Q = 1.74 (*n* = 60/3). *Cystidioles* lageniform or flexuous, hyaline, thin-walled, some with guttules, 8.7–33.5 × 2.7–5.6 μm. *Hymenial setae* subulate, few-lobed or sinuous, rarely branched, acute at the apex, often bi-radicated, dark brown, thick-walled, occasionally septate, 20.0–46.2 × 4.5–9.2 μm.

*Ecology/Substrate/Host*: Causes a white rot on dead trunks or branches of angiosperm trees, including *Carpinus laxiflora* and *Quercus*, in temperate forests at a wide range of *ca*. 30 to 900 m in altitude.

*Distribution*: Republic of Korea.

*Additional specimens examined:* See Additional file 6: Data S3.

*Notes*: *Fuscoporia australiana* is phylogenetically close to *F. koreana*, but has aseptate skeletal hyphae, smaller pores with 7–9 pores per mm, and larger basidia of 12–16 × 4–6 μm (Chen et al. 2020).* Fuscoporia koreana* is also phylogenetically closely related and morphologically similar to *F. gilva*, but *F. gilva* can be differentiated by the larger basidiospores, 4–5 × 3.0–3.5 μm (Dai 2010). *Fuscoporia koreana* and *F. semicephala* were both found in the Republic of Korea and are phylogenetically very closely related. *Fuscoporia semicephala* may be distinguished by aseptate skeletal hyphae, larger basidia of 9.2–14.2 × 4.5–6.9 μm, and the wider basidiospores, 4.0–4.8 × 2.8–3.4 μm (Q = 1.41).

***Fuscoporia reticulata*** Y. Cho, D. Kim & Y. W. Lim, **sp. nov.** (Fig. 6).

MycoBank: MB 844766.

*Etymology*: ‘reticulata’ refers to the reticulate hymenophore.

*Type*: **Republic of Korea**: Gyeonggi-do, Yongmun-myeon, Yangpyeong-gun, Sinjeom-ri, 37°33′31.2ʹʹ N 127°35′48.0ʹʹ E, 549 m, Mt. Jungwon, mixed forest, on angiosperm branch, 15 Jan 2016, *Young Woon Lim*, *Nam Kyu Kim*, *Hyun Lee*, *Hae Jin Cho*, *Seobihn Lee*, & *Vladimir Li* (SFC20160115-16—holotype).

*Diagnosis*: Basidiomes perennial, resupinate, found in temperate regions; pores 4–5 per mm; mycelial setae abundant, 66.9–217.2 × 6.3–10.0 μm; hymenial setae of two types, long and narrowly subulate or relatively short and ventricose, straight but usually bent near the base, occasionally septate.

*Description*: *Basidiome* perennial, resupinate, to 1.5 mm thick at center. *Pore surface* rusty brown (6D4), margin sterile, 0.8–1 mm wide, paler than pore surface. *Pores* reticulate, irregular, sinuous, 4–5 pores per mm. *Tubes* concolor with pore surface, corky, to 1 mm deep, dissepiments to 0.3 mm thick, entire, easily cracked when dry, abundant hymenial setae seen under stereomicroscope. *Subiculum* olive brown (4D8) to dark brown (5E8), corky, to 0.6 mm thick.

*Hyphal system* dimitic; generative hyphae hyaline to pale yellow, thin-walled, branched, simple septate, 1.7–2.5 μm wide in tube, 1.9–2.8 μm wide in subiculum, some at dissepiment edge encrusted with crystals; skeletal hyphae dominant in both tubes and subiculum, more loose in subiculum, rusty brown to golden yellow, thick-walled with medium lumen, unbranched, interwoven, 2–3 secondary septa present at the apex, 2.3–3.7 μm wide in tube, 2.3–3.4 μm wide in subiculum.

*Basidia* clavate, some of them slightly swollen on one side, four sterigmata, simple septum at the base, 9.0–11.8 × 3.6–5.7 μm; basidioles smaller in size compared to basidia. *Basidiospores* ellipsoid, hyaline, thin-walled, smooth, occasionally with 1–3 small guttules, IKI–, CB–, (3.9–)4.1–4.8(–5.1) × (2.3–)2.5–3.0(–3.1) μm, L = 4.46 μm, W = 2.80 μm, Q = 1.59 (*n* = 40/2). *Cystidioles* not seen. *Hymenial setae* of two types, long and narrowly subulate or relatively short and ventricose, straight but usually bent near the base, acute at the apex, dark brown, thick-walled, occasionally septate, 21.9–81.2 × 7.5–11.8 μm. *Mycelial setae* abundant in subiculum, acute or somewhat obtuse at the apex, dark brown, thick-walled, aseptate, 66.9–217.2 × 6.3–10.0 μm.

*Ecology/Substrate/Host*: On branches of angiosperms in temperate forests, causing a white rot.

*Distribution*: Republic of Korea.

*Additional specimens examined:* See Additional file 6: Data S3.

*Notes*: *Fuscoporia reticulata* is phylogenetically closely related to *F. monticola*, *F. septiseta*, and *F. sinica*. *Fuscoporia monticola* and *F. septiseta* differ from *F. reticulata* in having annual basidiomes with larger pores (2–3 pores per mm), and larger basidia; 15–20 × 4.5–6.2 μm in *F. monticola* and 17–20 × 4.8–7.0 μm in *F. septiseta* (Chen et al. 2019). *Fuscoporia septiseta* also differs in the known geographical distribution, as it is found in North America. *Fuscoporia sinica* differs from *F. reticulata* in having cylindrical basidiospores (Q = 2.32–2.38) that are larger, 5.8–7.0 × 2.4–3.0 μm (Chen et al. 2019).

***Fuscoporia semicephala*** Y. Cho, D. Kim & Y. W. Lim, **sp. nov.** (Fig. 7).

MycoBank: MB 844767.

*Etymology*: ‘semicephala’, after the half-pileate basidiome of the species.

*Type*: **Republic of Korea**: Jeollanam-do, Goheung-gun, Yeongnam-myeon, Ucheon-ri, 34°37′10.8ʹʹ N 127°26′09.3ʹʹ E, 438 m, Palyeongsan Nature Recreation Forest, mixed forest, on *Quercus* sp., 24 May 2017, *Jae Young Park* (SFC20170524-08—holotype).

*Diagnosis*: Basidiomes perennial, effused-reflexed to pileate, found in temperate regions in Asia; pores 5–7 per mm; hymenial setae subulate to ventricose, often bi-radicated, reddish brown, some septate, 18.0–34.8 × 5.0–8.7 μm; basidiospores ellipsoid to ovoid, occasionally with 1–3 small guttules, 4.0–4.8 × 2.8–3.4 μm.

*Description*: *Basidiome* perennial, effused-reflexed to pileate, solitary to imbricate. *Pileus* dimidiate, undulate, laterally fused, projecting 0.8–1.5 cm, to 6.7 cm wide and to 0.9 cm thick at base. *Pileal surface* concentrically zonate, glabrous, sometimes nodulose, pale brown (5C4); margin obtuse, pale yellowish grey (1B1) to dark beige (4C4), to 1 mm. *Pore surface* pale brown (5D4), margin sterile, 1–3 mm wide, light olive (3B3). *Pores* circular, sometimes sinuous or irregular, 5–7 pores per mm. *Tubes* pale yellow (5A2), corky, to 3.0 cm deep, dissepiments to 0.1 mm thick, entire, abundant hymenial setae seen under stereomicroscope. *Context* yellowish orange (5A4) to tan (6C6) near the tube, corky, to 4.4 cm thick.

*Hyphal system* dimitic; generative hyphae hyaline to pale yellow, thin-walled, branched, simple septate, 1.7–2.8 μm wide in tube, 2.1–2.8 μm wide and rare in context, some at dissepiment edge coarsely encrusted with stellate crystals; skeletal hyphae dominant in both context and tubes, rusty brown to golden brown, thick-walled with medium to wide lumen, unbranched, 2–3 secondary septa present at the apex, 2.9–4.2 μm wide in tube, 3.2–4.8 μm wide in context.

*Basidia* clavate, some of them slightly swollen on one side, four sterigmata, simple septum at the base, 9.2–14.2 × 4.5–6.9 μm; basidioles about the same size as basidia. *Basidiospores* ellipsoid to ovoid, hyaline, thin-walled, smooth, occasionally with 1–3 small guttules, IKI–, CB–, (3.8–)4.0–4.8(–5.1) × (2.6–)2.8–3.4(–3.7) μm, L = 4.36 μm, W = 3.10 μm, Q = 1.41 (*n* = 60/2). *Cystidioles* frequent, fusoid, lageniform, or cylindric-flexuous, hyaline, thin-walled, 14.4–32.8 × 3.3–5.3 μm. *Hymenial setae* subulate to ventricose, acute to acuminate at the apex, often bi-radicated, reddish brown, thick-walled, some septate, 18.0–34.8 × 5.0–8.7 μm.

*Ecology/Substrate/Host*: On angiosperm trees in temperate forests, causing a white rot.

*Distribution*: Republic of Korea.

*Additional specimens examined***:** See Additional file 6: Data S3.

*Notes*: *Fuscoporia australiana* and *F. gilva* are morphologically similar and closely related to *F. semicephala*. *Fuscoporia australiana* may be differentiated by the smaller pores with 7–9 pores per mm and larger basidia, 12–16 × 4–6 μm (Chen et al. 2020). *Fuscoporia gilva* differs from *F. semicephala* in having septate skeletal hyphae (Dai 2010).

## DISCUSSION

Numerous misidentified GenBank ITS sequences were encountered in making this study, either unpublished or differing from the annotations in the research articles (Additional file 3: Table S2). These misidentifications may have arisen in any of several ways. Experimental laboratory contamination, failure of the sequence authors to perform basic quality control on the generated sequences (Nilsson et al. 2012), uncritical BLAST-based identification (Hofstetter et al. 2019), and late or no follow-up taxonomic revisions are a few possibilities. For the *Fuscoporia* sequences that disagree between GenBank and published articles, mislabelling or swapping sequences during mass sequence uploads to the public database may explain paraphyletic clades (e.g. *F. chinensis* and *F. plumeriae* sequences). Another possibility is the identification of specimens based solely on morphological characters before the emergence of new species from East Asia based on molecular analyses (Chen et al. 2020). At that time, the availability of morphological descriptions for reference was limited to a few European species, such as *F. ferruginosa* and *F. gilva* (Dai 1999, 2010; Jang et al. 2016). As such, many *Fuscoporia* specimens, such as *F. chinensis*, with effused-reflexed to pileate basidiomes, setaceous to nodulous pileal surfaces, presence of cystidioles, and ellipsoid to cylindric basidiospores have been recorded as *F. gilva*, which has been recognized as common worldwide. However, multi-marker phylogenetic analyses have revealed that *F. gilva* is a different species from *F. chinensis*, and that the two species are also divided by geographical distribution (Chen et al. 2020).

There was a significant proportion of unidentified sequences (29.8%), such as ‘*Fuscoporia* sp.’ and ‘Uncultured fungus,’ that were identified to the species level when grouped with type-derived sequences in the phylogenetic analyses (Fig. 1 and Additional file 3: Table S2). Unidentified sequences in the species complexes, such as the majority in section II, were not determined. Specimens associated with these unidentified sequences require more extensive analyses using multi-marker, morphological, and ecological data. Five of the twelve species complexes, namely *F. australiana*/*F. bambusae*/*F. dolichoseta* (Complex III), *F. ramulicola*/*F. subferrea* (Complex IV), *F. gilvoides*/*F. karsteniana* (Complex VI), *F. chinensis*/*F. semicephala* (Complex VII), and *F. rhabarbarina*/*F. senex* (Complex XI), were clearly resolved in the multi-marker analyses (Fig. 2). This indicates that evaluating ITS alone is insufficient to differentiate and identify some *Fuscoporia* species because ITS has low resolution, which would explain a ‘*Fuscoporia* sp.’ annotation.

The multi-marker analyses provided only a partial answer to *Fuscoporia* species differentiation. Two species complex pairs in the ITS tree, *F*. *bambusicola*/*F. latispora* (Complex I), and *F. monticola*/*F. sinica* (Complex II), remained monophyletic in the multi-marker phylogenetic tree. Individual species in both pairs have been addressed thoroughly as distinct species based on their morphological characters and geographical distribution. *Fuscoporia monticola* differs from *F. sinica* in that it occasionally has simple-septate mycelial setae and is distributed in southern China. In contrast, *F. sinica* has aseptate mycelial setae and longer basidiospores, and occurs in north western China (Chen et al. 2019). For the *F*. *bambusicola*/*F. latispora* pair, *F. latispora* has a darker sterile margin than the pore surface due to locally abundant mycelial setae compared to *F. bambusicola* and is found on angiosperm wood in Central America (Chen et al. 2019). In contrast, *F. bambusicola* grows on bamboo and is distributed throughout southern China (Chen et al. 2020). However, despite the aforementioned factors that separate these species, it was difficult to confirm that each pair was truly different, as they were short in sample numbers and sequences. To clearly distinguish between them, more specimens should be collected to validate the differences in morphology and geographical distribution. Conducting a mating test may also confirm the separation of molecularly indifferentiable species. Mating compatibility tests may verify the intersterility between morphologically similar or phylogenetically closely related species from different geographical locations (Fischer and Binder 1995; Bao et al. 2004).

Ecological features such as geographical distribution and habitat often serve as aids to species differentiation or identification in *Fuscoporia*. Restricted geographical distribution of *Fuscoporia* species has been frequently reported. For example, *Fuscoporia chrysea* (particularly in the neotropics) and *F. palmicola* have only been reported in Central America (Bondarceva et al. 1992; Baltazar and Gibertoni 2010), whereas *F. atlantica*, *F. licnoides*, and *F. marquesiana* have only been reported in Brazil (Pires et al. 2015; Yuan et al. 2020). Additionally, new species have been reported solely based on morphological characters, such as *F. bifurcata* (Baltazar et al. 2009) and *F. valenzuelae* (Raymundo 2021). However, ecological traits and morphological characters are often not discriminatory among similar or closely related species. Therefore, it is imperative to evaluate species phylogenetically through multi-marker analyses and, if possible, biologically through mating compatibility tests. In brief, an integrative taxonomic approach, considering different combinations of biological, ecological, phenetic, and/or phylogenetic characters (Chethana et al. 2021), is essential for the most accurate species differentiation and identification of *Fuscoporia*, as numerous species demand more than one species recognition criterion to distinguish them.

Multi-marker phylogenetic analyses of ITS + nrLSU + *rpb2* + *tef1* with 52 newly assessed specimens from this study revealed five previously undescribed species of *Fuscoporia*: *F. dolichoseta*, *F. gilvoides*, *F. koreana*, *F. reticulata*, and *F. semicephala.* These new species, all with perennial basidiomes, were found in temperate regions. All were also well supported as novel ecologically, phylogenetically, and morphologically, but some micro-morphological characters overlapped with other closely related species. Notably, *F. dolichoseta* formed a monophyletic clade with *F. australiana*/*F. bambusae*/*F. ferruginosa*/*F. plumeriae* in species complex III (Fig. 1B). All five species were well differentiated by geographical and ecological characters, as well as by multi-marker analyses (Fig. 2). *Fuscoporia australiana* and *F. plumeriae* have been reported in Australia and *F. plumeriae* in Singapore (Chen et al. 2020), whereas *F. dolichoseta* has so far only been reported in the Republic of Korea. The type country for *F. ferruginosa* is the USA. *Fuscoporia bambusae* grows on *Bambusaceae* (Chen et al. 2020), whereas *F. dolichoseta* grows on angiosperms. *Fuscoporia bambusae* inhabits tropical areas, such as Thailand, whereas *F. dolichoseta* has only been found in temperate regions.

*Fuscoporia* species are actively assessed for their metabolites for applications in nutraceuticals, with *F. gilva* and *F. torulosa* having received the most attention. Various anticancer and anticholinesterase agents have been detected in *F. torulosa*, which display antibacterial, antifungal, antioxidant, and cytotoxic activities (Covino et al. 2019; Deveci et al. 2019). *Fuscoporia gilva* also exhibits various bioactivities, displaying potential for diabetes control and improved immunity (Sun et al. 2020; Duong and Dang 2022). However, *F. gilva* has been reported as “*Phellinus gilvus*” in some studies, despite the species combination of Wagner and Fischer (2002). Furthermore, it is difficult to determine whether all studied materials were truly *F. gilva* or if they were recently reported new species, such as *F. gilvoides*. This is because there is no type-derived sequence available, and research articles rarely indicate how they identified the species or do not provide the molecular data they used for identification. There are also studies that have used unidentified strains because of insufficient assessment for species identification.

Some strains in published articles have been revised with respect to their identities through phylogenetic analysis. For example, *F. gilva* KUC20121123-27 (accession KJ668544 for ITS) in a diversity study by Jang et al. (2016) was revised to *F. koreana* (Fig. 1). There were several cases where fungal study materials in published papers were re-identified by a third party (Stockinger et al. 2009; Fernández-López et al. 2018), although the re-identification was lost or disregarded due to the unrevised primary deposit. Subsequent research papers that utilized misidentified study material further accumulated misconceived information. To obtain an accurate chemical or diversity profile for each species, it is important to identify the materials studied using high-precision methods and inspect for misidentified or misleading references (Wasser 2011). Incorrect and insufficient information in taxonomic studies (Durkin et al. 2020) causes confusion and accumulates further misleading data in the databases.

Regardless of the research area, studies of *Fuscoporia* species could avoid incorrectly annotated public sequences for species identification through several practices. BLAST may be the easiest and fastest method for species identification; however, BLAST conducted for all *Fuscoporia* ITS sequences in this study revealed 14.0% (91 of 649, excluding the newly generated accessions in this study) with unmatching results for the top five best hits, and 30.2% (196/649) of query sequences were not identified to the species level (Additional file 4: Table S3). For the BLAST result, and as far as possible, it is essential to identify the query sequence based on sequences derived from type materials [many are listed under the Fungal Internal Transcribed Spacer RNA (ITS) RefSeq Targeted Loci Project, PRJNA177353]. Occasionally, type materials are not indicated as a “type” in GenBank and are only available in the respective research articles. In the absence of type materials, species identification based on the accessions listed in the most recently published taxonomy papers may be the most plausible approach. Using a more comprehensive database, such as UNITE, may further reduce the use of unverified sequences as a reference (Abarenkov et al. 2010). In addition to BLAST, validating species identity using a phylogenetic approach may ensure greater confidence. Similarly, comparing the morphological characters of the studied material with the descriptions of the type specimens in taxonomic papers may also be helpful. For taxonomists, it is crucial to follow-up on taxonomic revisions and update GenBank records as a primary uploader, as only the uploader may edit the annotations. Reviewers and journal editors similarly have a role in enforcing journal policies and sound levels of data annotation and persistency. As in this study, a third-party user may request and even implement a revision in records in secondary databases such as UNITE. Although it is a demanding and never-ending process, a systematic administration of primary public databases is indispensable for proceeding towards an unquestionable scientific community.

## CONCLUSIONS

Molecular analysis is essential for identifying *Fuscoporia* species, as the morphological characters of these crust fungi are often indistinct. Even then, a cautious approach is required when using ITS alone for species identification and phylogenetic studies of *Fuscoporia*, as ITS has a low resolution for species differentiation, and there are many incorrectly annotated sequences in GenBank. Assessment of *Fuscoporia* species with multiple genetic regions has increased the resolution of species differentiation and has led to the discovery of five new species. Five new species were described in this study using attentive taxonomic identification approaches. In addition to the phylogenetic approach, aspects such as biogeographical distribution and mating tests may also aid in differentiating and identifying closely related species. It is vital that researchers accurately identify species for future applications. We hope that the provision of multi-marker sequences, morphological descriptions of the new species, and revision of GenBank *Fuscoporia* ITS sequences based on type- or type locality-derived sequences and published reliable sequences in this study will serve as supportive data for further analyses in various research areas that require sensitive species identification.

## Supplementary Information

**Additional file 1.** ITS sequence alignments of all assessed GenBank accessions.

**Additional file 2.** UNITE submission records of species reannotation for GenBank ITS accessions for *Fuscoporia* species.

**Additional file 3.** Topmost hit from BLASTn results for all *Fuscoporia* ITS sequence accessions.

**Additional file 4.** Top five hits from BLASTn results for all *Fuscoporia* ITS sequence accessions.

**Additional file 5.** Concatenated multi-marker (ITS + nrLSU + *rpb2* + *tef1*) sequence alignments of all assessed GenBank records.

**Additional file 6.** List of additional specimens examined for the five new *Fuscoporia* species.
