# Supplementary material for: Validation of Fuscoporia (Hymenochaetales, Basidiomycota) ITS sequences and five new species based on multi-marker phylogenetic and morphological analyses
Source: IMA Fungus. 2023 Jun 28;14:12. doi: 10.1186/s43008-023-00117-6 (PMC10308793; doi:10.1186/s43008-023-00117-6)
Supplement: Supplementary file 6 — Additional file 6. List of additional specimens examined for the five new Fuscoporia species. [file 43008_2023_117_MOESM6_ESM.docx]

**Taxonomy (Extended)**

***Fuscoporia dolichoseta*** Y. Cho, D. Kim & Y. W. Lim, sp. nov.

**Additional specimens examined:** Republic of Korea: Jeollabuk-do, Jinan-gun, Jeongcheon-myeon, Garyong-ri, 35°53'37.8"N 127°25'40.7"E, 357 m, Mt. Unjang (Natural Recreational Forest); 23 Jul 2014, Young Woon Lim (SFC20140723-58). Republic of Korea: Gangwon-do, Inje-gun, Sangnam-myeon, 37°52'24"N 128°18'53.3"E, 381 m, Mt. Bangtae; 12 Feb 2015, Jae young Park (SFC20150212-09). Republic of Korea: Gangwon-do, Pyeongchang-gun, Jinbu-myeon, 37°43'55.8"N 128°35'40.3"E, 668 m, *Abies holophylla* trail in Mt. Woljeong, on dead trunk of angiosperm; 06 Oct 2016, Jae Young Park, Nam Kyu Kim (SFC20161006-16). Republic of Korea: Gangwon-do, Taebaek-si, Sodo-dong, 37°06'51.6"N 128°56'58.2"E, 908 m, Mt. Taebaek Danggol Valley, on dead trunk of angiosperm; 31 Jul 2019, Jae Young Park, Abel Lupalpa, Shinnam Yoo (SFC20190731-26). Republic of Korea: Gangwon-do, Taebaek-si, Sodo-dong, 37°06'50.2"N 128°56'59.3"E, 923 m, Mt. Taebaek Danggol Valley, on dead trunk of angiosperm; 22 Aug 2019, Jae young Park (SFC20190822-16).

***Fuscoporia gilvoides*** Y. Cho, D. Kim & Y. W. Lim, sp. nov.

**Additional specimens examined:** Republic of Korea: Chungcheongnam-do, Gongju-si, Useong-myeon, Hancheon-ri; 36°33'56.4"N 127°04'23.2"E, 349 m, Mt. Museong, on *Prunus*; 26 Jul 2012, Young Woon Lim (SFC20120726-09). Republic of Korea: Jeollabuk-do, Jinan-gun, Jeongcheon-myeon, Garyong-ri; 35°53'37.8"N 127°25'40.7"E, 357 m, Mt. Unjang (Natural Recreational Forest); 11 Apr 2014, Young Woon Lim (SFC20140411-21). Republic of Korea: Jeollabudpdo, Jinan-gun, Donghyang-myeon, Seongsan-ri; 35°49'25.8"N 127°31'59.2"E, 275 m, Mt. Cheonban, on dead trunk of angiosperm; 11 Sep 2014, Young Woon Lim (SFC20140911-32). Republic of Korea: Incheon, Ganghwa-gun, Hwado-myeon, Heungwan-ri; 37°37'00.9"N 126°24'17.7"E, 107 m, Mt. Mani, on dead trunk of *Quercus*; 26 Jun 2015, Young Woon Lim (SFC20150626-01). Republic of Korea: Jeju-do, Namjeju-gun, Namwon-eup; 33°18'08.0"N 126°35'29.8"E, 222 m, Warm Temperate and Subtropical Forest Research Center; 02 Jul 2015, Young Woon Lim (SFC20150702-23). Republic of Korea: Gyeongsangbuk-do, Ulleung-gun, Buk-myeon, Nari 1-gil; 37°31'31.7"N 130°52'11.5"E, 413 m, Naribunji Basin, on trunk of dead angiosperm; 20 Oct 2015, Young Woon Lim (SFC20151020-22). Republic of Korea: Gyeongsangbuk-do, Ulleung-gun, Buk-myeon, Nari 1-gil; 37°31'31.7"N 130°52'11.5"E, 413 m, Naribunji Basin, on angiosperm in mixed forest; 14 Jun 2016, Young Woon Lim (SFC20160614-39). Republic of Korea: Incheon, Ongjin-gun, Yeongheung-myeon; 37°15'54.9"N 126°27'44.0"E, 92 m, Yeongheong-do Island, on dead trunk of *Quercus mongolia* in angiosperm forest; 21 Jun 2016, Nam Kyu Kim, Jae Young Park (SFC20160621-12). Republic of Korea: Incheon, Ongjin-gun, Bukdo-myeon; 37°32'32.2"N 126°25'31.1"E, 36 m, Sido island, on dead trunk of *Quercus mongolia* in angiosperm forest; 29 Jun 2016, Nam Kyu Kin, Jae Young Park, Myung Soo Park (SFC20160629-33). Republic of Korea: Incheon, Ongjin-gun, Bukdo-myeon; 37°31'50.0"N 126°27'24.2"E, 21 m, Sindi island, on dead trunk of angiosperm in mixed forest; 12 Jun 2016, Nam Kyu Kin, Jae Young Park, Myung Soo Park (SFC20160712-45). Republic of Korea: Jeju-do, Jeju-si, Jocheon-eup, Gyorae-ri; 33°26'32.4"N 126°40'00.1"E, 435 m, Gyorea Natural Recreation Forest, on trunk of dead angiosperm; 14 Jul 2016, Jae Young Park, Myung Soo Park (SFC20160714-16). Republic of Korea: Incheon, Ongjin-gun, Bukdo-myeon, Sido-ri; 37°32'26.1"N 126°20'12.0"E, 17 m, Jangbongdo island; 26 Jun 2016, Nam Kyu Kim, Jae Young Park (SFC20160726-41). Republic of Korea: Gyeonggi-do, Guri-si, Donggureung-dong; 37°37'13.6"N 127°07'40.3"E, 66 m, Royal tombs, on trunk of angiosperm in mixed forest; 12 Aug 2016, Nam Kyu Kim, Hyun Lee, Vladimir Li (SFC20160812-23). Republic of Korea: Incheon, Ongjin-gun, Yeongheung-myeon; 37°15'54.9"N 126°27'44.0"E, 92 m, Yeongheong-do Island, on dead trunk of *Quercus acutissima* in angiosperm forest; 22 Nov 2016, Jae Young Park, Nam Kyu Kim (SFC20160922-07). Republic of Korea: Incheon, Ongjin-gun, Yeongheung-myeon; 37°15'54.9"N 126°27'44.0"E, 92 m, Yeongheong-do Island, on dead trunk of *Quercus* in mixed forest; 22 Nov 2016, Jae Young Park, Nam Kyu Kim (SFC20160922-14). Republic of Korea: Seoul, Jongno-gu, Jong-ro; 37°34'30.5"N 126°59'38.8"E, 41 m, Royal Ancestral Shrine, on dead trunk of *Quercus* in mixed forest; 23 Nov 2016, Hyun Lee, Seung-Yoon Oh, Vladimir Li (SFC20160923-40). Republic of Korea: Gyeonggi-do, Goyang-si, Deogyang-gu, Yongdu-dong; 37°37'35.5"N 126°53'53.2"E, 39 m, Royal tombs, on dead trunk of *Quercus dentata* in angiosperm forest; 12 Oct 2016, Hyun Lee, Suldbold Jargalmaa (SFC20161012-20). Republic of Korea: Seoul, Gwanak-gu, Sillim-dong; 37°26'56.3"N 126°57'08.9"E, 231 m, Mt. Gwanak; 05 Nov 2018, Min Ji Kim, Ki Hyung Park (SFC20180905-15). Pakistan: KP province, Swat district, Chinar Jambil; 34°41′53″N, 72°27′58″E, on *Quercus dilatata* in deciduous forest; Shahid Hussain (MUGBt). Pakistan, KP province, district Swat, Miandam; 35°3′15″N, 72°35′57″E, on *Abies pindrow* in mixed-conifer forest; Shahid Hussain (MUKM-2).

***Fuscoporia koreana*** Y. Cho, D. Kim & Y. W. Lim, sp. nov.

**Additional specimens examined:** Republic of Korea: Gyeonggi-do, Guri-si, Donggureung-ro; 37°37'11.9"N 127°07'53.4"E, 39 m, Royal tombs, on fallen branch of angiosperm; 26 June 2014, Young Woon Lim (SFC20140626-25). Republic of Korea: Gangwon-do, Donghae-si, Samhwa-dong; 37°27'55.3"N 129°01'07.4"E, 259 m, Mureung Valley, on dead trunk of angiosperm; 12 Jun 2015, Young Woon Lim (SFC20150612-02). Republic of Korea: Gyeonggi-do, Gwachoen-si, Makgye-dong; 37°25'03.7"N 127°02'31.5"E, 493 m, Mt. Chenggye, on dead trunk of *Quercus*; 25 June 2015, Young Woon Lim (SFC20150625-05). Republic of Korea: Gyeonggi-do, Gwachoen-si, Makgye-dong; 37°25'03.7"N 127°02'31.5"E, 493 m, Mt. Chenggye, on dead trunk of *Quercus*; 25 June 2015, Young Woon Lim (SFC20150625-07). Republic of Korea: Gyeonggi-do, Gwangju-si, Docheok-myeon, Yujeong-ri; 37°17'48.7"N 127°18'11.8"E, 420 m, Hiking trail, on dead trunk of *Quercus*; 25 June 2015, Young Woon Lim (SFC20150625-30). Republic of Korea: Gyeonggi-do, Uiwang-si, Cheonggye-ro; 37°24'45.6"N 127°02'06.3"E, 293 m, Mt. Cheggye (Chungkeisa), on dead trunk of angiosperm; 20 Aug 2015, Young Woon Lim (SFC20150820-60). Republic of Korea: Seoul, Gwanak-gu, Sillim-dong; 37°26'38.8"N 126°57'38.5"E, 449 m, Mt. Gwanak, on dead trunk of angiosperm; 14 Sep 2015, Young Woon Lim (SFC20150914-16). Republic of Korea: Incheon, Ongjin-gun, Bukdo-myeon, Jangbongdo island; 37°32'28.5"N 126°20'08.9"E, 34 m, mixed forest, on dead trunk of *Quercus mongolia*; 06 Sep 2016, Jae Young Park, Nam Kyu Kim (SFC20160906-18). Republic of Korea: Gyeongsangnam-do, Hapcheon-gun, Gaya-myeon; 35°49'53.1"N 128°04'53.7"E, 911 m, Mt. Gaya (Haeinsa), on fallen branch of angiosperm; 08 Sep 2017, Hae Jin Cho, Ki Hyung Park, Namhwi Kim (SFC20170908-74). Republic of Korea: Gangwon-do, Hongcheon-gun; 37°44'29.0"N 128°04'04.4"E, 421 m, on dead trunk; 19 Oct 2017, Nam Kyu Kim (SFC20171019-11). Republic of Korea: Gangwon-do, Hongcheon-gun, Hongcheon-eup, Sammachi-ri; 37°36'38.4"N 127°53'52.6"E, 321 m, on angiosperm; 12 Dec 2017, Nam Kyu Kim (SFC20171212-32). Republic of Korea: Gangwon-do, Hongcheon-gun, Hongcheon-eup, Sammachi-ri; 37°36'38.4"N 127°53'52.6"E, 321 m, on angiosperm; 12 Dec 2017, Nam Kyu Kim (SFC20171212-42). Republic of Korea: Gangwon-do, Hongcheon-gun, Hongcheon-eup, Sammachi-ri; 37°36'38.4"N 127°53'52.6"E, 321 m, on angiosperm; 14 Dec 2017, Nam Kyu Kim (SFC20171214-10). Republic of Korea: Gyeongsangnam-do, Hapcheon-gun, Gaya-myeon; 35°48'10.2"N 128°05'50.1"E, 625 m, Mt. Gaya (Baekryeonam), on fallen branch of *Carpinus laxiflora*; 25 Jul 2018, Hyun Lee, Hae Jin Cho, Namhwi Kim (SFC20180725-17). Republic of Korea: Seoul, Gwanak-gu, Sillim-dong; 37°26'38.8"N 126°57'38.5"E, 449 m, Mt. Gwanak, on dead stump of *Quercus*; 18 Aug 2018, Young Woon Lim, Hyun Lee, Hye Jin Cho, Ki Hyung park, Abel Lupalpa, (SFC20180818-19). Republic of Korea: Seoul, Gwanak-gu, Sillim-dong; 37°27'32.2"N 126°56'47.9"E, 99 m, Mt. Gwanak Lake Park; 05 Sep 2018, Myung Soo Park, Jaya Seelan Sathiya Seelan, Namhwi Kim (SFC20180905-56). Republic of Korea: Gyeongsangbuk-do, Bonghwa-gun, Seokpo-myeon, Daehyeon-ri; 37°4′28.2108"N, 128°57′31.734"E, 629 m, Baekchoen valley, on dead trunk of angiosperm; 22 Mar 2019, Young Woon Lim, Myung Soo Park, Min Ji Kim, Hyun Lee, Ki Hyung Park, Shinnam Yoo, Namhwi Kim (SFC20190322-27).

***Fuscoporia reticulata*** Y. Cho, D. Kim & Y. W. Lim, sp. nov.

**Additional specimens examined:** Republic of Korea: Chungcheongnam-do, Yongmun-myeon, Seosan-si, Unsan-myeon, Yonghyeon-ri; 36°45'01.2"N 126°36'19.1"E, 290 m, mixed forest, on angiosperm branch; 10 Oct 2012, Young Woon Lim, Jae Young Park, Young Ju Min (SFC20121010-19).

***Fuscoporia semicephala*** Y. Cho, D. Kim & Y. W. Lim, sp. nov.

**Additional specimens examined:** Republic of Korea: Jeollanam-do, Goheung-gun, Yeongnam-myeon, Ucheon-ri; 34°37'27.5"N 127°26'07.6"E, 427 m, Palyeongsan Nature Recreation Forest, mixed forest, on angiosperm; 12 Jul 2017, Jae Young Park, Komsit Wissitrassameewong (SFC20170712-20).
